# Cost-effectiveness of antenatal multiple micronutrients and balanced energy protein supplementation compared to iron and folic acid supplementation in India, Pakistan, Mali, and Tanzania: A dynamic microsimulation study

**DOI:** 10.1371/journal.pmed.1003902

**Published:** 2022-02-22

**Authors:** Nicole Young, Alison Bowman, Kjell Swedin, James Collins, Nathaniel D. Blair-Stahn, Paulina A. Lindstedt, Christopher Troeger, Abraham D. Flaxman

**Affiliations:** Institute for Health Metrics and Evaluation, Seattle, Washington, United States of America; University of Manchester, UNITED KINGDOM

## Abstract

**Background:**

Malnutrition among women of childbearing age is especially prevalent in Asia and sub-Saharan Africa and can be harmful to the fetus during pregnancy. In the most recently available Demographic and Health Survey (DHS), approximately 10% to 20% of pregnant women in India, Pakistan, Mali, and Tanzania were undernourished (body mass index [BMI] <18.5 kg/m^2^), and according to the Global Burden of Disease (GBD) 2017 study, approximately 20% of babies were born with low birth weight (LBW; <2,500 g) in India, Pakistan, and Mali and 8% in Tanzania. Supplementing pregnant women with micro and macronutrients during the antenatal period can improve birth outcomes. Recently, the World Health Organization (WHO) recommended antenatal multiple micronutrient supplementation (MMS) that includes iron and folic acid (IFA) in the context of rigorous research. Additionally, WHO recommends balanced energy protein (BEP) for undernourished populations. However, few studies have compared the cost-effectiveness of different supplementation regimens. We compared the cost-effectiveness of MMS and BEP with IFA to quantify their benefits in 4 countries with considerable prevalence of maternal undernutrition.

**Methods and findings:**

Using nationally representative estimates from the 2017 GBD study, we conducted an individual-based dynamic microsimulation of population cohorts from birth to 2 years of age in India, Pakistan, Mali, and Tanzania. We modeled the effect of maternal nutritional supplementation on infant birth weight, stunting and wasting using effect sizes from Cochrane systematic reviews and published literature. We used a payer’s perspective and obtained costs of supplementation per pregnancy from the published literature. We compared disability-adjusted life years (DALYs) and incremental cost-effectiveness ratios (ICERs) in a baseline scenario with existing antenatal IFA coverage with scenarios where 90% of antenatal care (ANC) attendees receive either universal MMS, universal BEP, or MMS + targeted BEP (women with prepregnancy BMI <18.5 kg/m^2^ receive BEP containing MMS while women with BMI ≥18.5 kg/m^2^ receive MMS). We obtained 95% uncertainty intervals (UIs) for all outputs to represent parameter and stochastic uncertainty across 100 iterations of model runs. ICERs for all scenarios were lowest in Pakistan and greatest in Tanzania, in line with the baseline trend in prevalence of and attributable burden to LBW. MMS + targeted BEP averts more DALYs than universal MMS alone while remaining cost-effective. ICERs for universal MMS compared to baseline IFA were $52 (95% UI: $28 to $78) for Pakistan, $72 (95% UI: $37 to $118) for Mali, $70 (95% UI: $43 to $104) for India, and $253 (95% UI: $112 to $481) for Tanzania. ICERs for MMS + targeted BEP compared to baseline IFA were $54 (95% UI: $32 to $77) for Pakistan, $73 (95% UI: $40 to $104) for Mali, $83 (95% UI: $58 to $111) for India, and $245 (95% UI: $127 to $405) for Tanzania. Study limitations include generalizing experimental findings from the literature to our populations of interest and using population-level input parameters that may not reflect the heterogeneity of subpopulations. Additionally, our microsimulation fuses multiple sources of data and may be limited by data quality and availability.

**Conclusions:**

In this study, we observed that MMS + targeted BEP averts more DALYs and remains cost-effective compared to universal MMS. As countries consider using MMS in alignment with recent WHO guidelines, offering targeted BEP is a cost-effective strategy that can be considered concurrently to maximize benefits and synergize program implementation.

## Abstract 2

In a dynamic microsimulation study, Nicole Young and colleagues compare the cost-effectiveness of antenatal multiple micronutrients and balanced-energy protein supplementation with iron and folic acid supplementation in India, Pakistan, Mali and Tanzania.

## Author summary

**Why was this study done?:**

- Iron and folic acid (IFA) supplementation and IFA-containing multiple micronutrient supplementation (MMS) taken during pregnancy can improve the health of newborns, particularly by increasing their birth weight, which reduces the risk of infant mortality.
- For pregnant mothers who are undernourished, increased caloric intake through balanced energy protein (BEP) supplementation can provide an additional benefit on birth weight and its associated health outcomes.
- We conducted this study to compare the costs and benefits of BEP, MMS, and IFA supplementation in areas burdened by low birth weight (LBW) and its associated health detriments.

**What did the researchers do and find?:**

- We estimated the impact of IFA, MMS, and BEP supplementation in India, Pakistan, Mali, and Tanzania by developing an individual-based dynamic microsimulation model of populations of newborns that tracked their health outcomes during the first 2 years of life.
- We found that supplying MMS to 90% of women who attend antenatal care (ANC) visits resulted in improving morbidity and mortality among children under 2 at a cost of $52 per disability-adjusted life year (DALY) saved for Pakistan, $72 for Mali, $70 for India, and $253 for Tanzania.
- Supplying BEP (containing MMS) to 90% of women who attend ANC with low body mass index (BMI) and MMS to 90% of women who attend ANC with adequate BMI saved more DALYs than supplying 90% of all women who attend ANC visits with MMS and was similarly cost-effective at $54 per DALY saved relative to existing IFA practice for Pakistan, $73 for Mali, $83 for India, and $245 for Tanzania.

**What do these findings mean?:**

- High coverage of MMS for women at ANC is a cost-effective strategy to improve child health outcomes relative to the existing coverage and practice of supplying only IFA.
- While supplying BEP (containing MMS) universally to women at ANC has the greatest impact on child health outcomes, targeting BEP for undernourished women and offering MMS to women of adequate BMI is more cost-effective and has greater impact on child health outcomes than supplying MMS alone.
- Our findings are limited by applying effects of maternal supplementation regimens from clinical trials to the modeled populations, and we did not model subnational variation.

## Introduction

Pregnancy requires increased energy and nutritional consumption to support the body’s changing physiology and fetal growth [1]. Without proper nourishment during pregnancy, the fetus may fail to reach adequate growth, resulting in intrauterine growth restriction (IUGR) [2]. IUGR can lead to babies born small for gestational age (SGA), defined as weight below the 10th percentile for gestational age or with low birth weight (LBW), defined as birth weight below 2,500 g. IUGR and LBW are associated with increased risk of neonatal death, infection, neurological impairment, wasting, and stunting in childhood as well as increased risk of chronic disease, diminished scholastic achievement, lower income, and decreased birth weight of offspring in adulthood [3,4]. Asia and Africa contain the greatest burden of LBW: In 2015, an estimated 48% and 24% of all affected newborns globally were from Asia and sub-Saharan Africa, respectively [5]. These regions also have the highest levels of micronutrient deficiencies among pregnant women and women of reproductive age [6]. Ensuring adequate maternal nutrition during gestation is a necessary and worthwhile investment that will have far-reaching benefits for current and future generations [7].

Supplementing women with micro and macronutrients during pregnancy can improve birth outcomes. Recently, the World Health Organization (WHO) updated its 2016 recommendation to use multiple micronutrient supplementation (MMS) that include IFA and additional 13 to 15 micronutrients in the context of rigorous research [8,9]. Cost-effectiveness analysis in some countries has shown that replacing IFA with MMS is highly cost-effective at under $100 per disability-adjusted life year (DALY) averted [10–13]. Currently, WHO recommends balanced energy protein (BEP) food supplements in settings with high prevalence of undernourished pregnant women (over 20%) [14]. BEP food supplements are foods in which protein provides less than 25% of the total energy content. They can come in several forms such as biscuits, beverages, or sachets and can be made using locally sourced ingredients [15]. The latest Cochrane review from 2015 found that use of BEP supplements during pregnancy can reduce the risk of stillbirth (relative risk [RR] = 0.6, 95% UI 0.39 to 0.94) and SGA (RR = 0.7, 95% UI 0.69 to 0.9) as well as increase mean birth weight (+40.96 g, 95% UI: 4.66 to 77.26) [15]. However, BEP’s potential may have been under realized in the trials due to heterogeneity in the BEP supplement compositions, supplementation period, and comparison groups used. Moreover, nonadherence or dietary substitution might have led to only small net increases in energy intake in some of the trials [15]. Greater effect sizes for BEP on birth weight have been reported by some studies, potentially due to study populations with larger energy deficits or use of a higher energy supplement [16–18]. Additionally, BEP may improve wasting and stunting in the first 5 years of life [19], but more studies are needed to draw conclusions on BEP’s long-term impacts on child growth [20]. Better optimized formulations of BEP that consist of equivalent micronutrients to MMS are available and may address these gaps [21]. These formulations create an integrated delivery vehicle that can improve acceptability and adherence and are currently under clinical trials.

One challenge to BEP implementation is its higher cost compared to IFA and MMS. One study estimated that BEP costs more than $500 per DALY averted, which may be over the affordability thresholds for some low- and middle-income countries [22]. A potentially more cost-effective strategy is to target BEP to undernourished pregnant women. Subgroup analysis comparing undernourished and adequately nourished women suggests a greater increase in birth weight among undernourished women (+66.96 g, 95% UI: 13.13 to 120.78) than adequately nourished women (+15.93 g, 95% UI: −20.83 to 52.69) [15]. Moreover, prevalence of obesity and overweight is increasing, and women with overweight or obesity are at higher risk of fetal overgrowth and macrosomia (≥4,000 g) [23]. Risks of supplementing high body mass index (BMI) women with BEP are still unknown [14]. Targeting BEP to undernourished women may be a cost-effective solution to maximize benefit. While targeting undernourished women for BEP does not fall under the 2016 WHO antenatal recommendation, we explored this approach in our simulation to understand its potential impact.

Maternal MMS and BEP supplementation are 2 of 10 critical direct nutritional interventions identified by the 2013 *Lancet* Series on maternal and child nutrition [24,25], but to the best of our knowledge, there is a lack of cost-effectiveness analysis comparing these interventions [26]. Leveraging estimates from the Global Burden of Disease (GBD) 2017 study, we used dynamic microsimulation to compare the impact on DALYs and the cost-effectiveness of supplementing pregnant women with (1) universal MMS; (2) universal BEP; or (3) MMS + targeted BEP (where undernourished women receive BEP supplements containing MMS with IFA and adequately nourished women receive MMS containing IFA) with current baseline coverage of IFA in India, Pakistan, Tanzania, and Mali. Although these countries have less than 20% prevalence of undernourished pregnant women, WHO criteria for universal BEP, they have high burdens of LBW, an outcome that BEP improves. Our model predicts the impact of several scenarios to inform government or donor decision-making on MMS and BEP supplementation for pregnant women.

## Methods

### Conceptual framework and model logic

**Fig 1:**
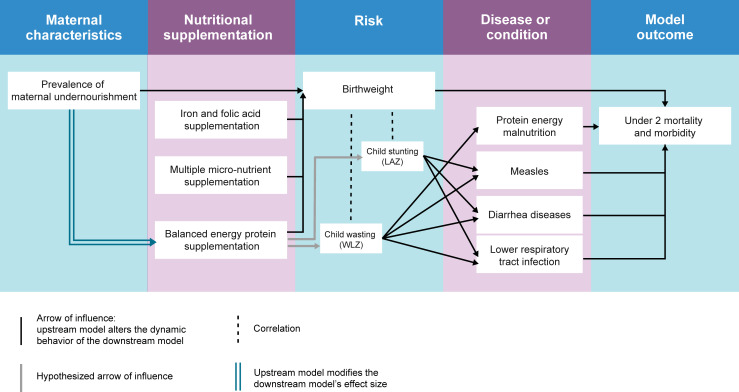
Model concept diagram. Logical causal flow of maternal characteristics and antenatal nutritional supplementation regimens on downstream effects and ultimate impact on mortality and morbidity in our microsimulation model. In GBD 2017, LBW is modeled to directly affect all-cause mortality among early (0 to 6 days) and late neonatal (7 to 27 days) age groups. Gray arrows denote effects with potential but inconclusive evidence. GBD, Global Burden of Disease; LAZ, length-for-age z-score; LBW, low birth weight; WLZ, weight-for-length z-score.

We developed an individual-based dynamic microsimulation model in Python 3.6 using the Vivarium simulation framework [27] to compare the DALYs averted and costs of various maternal supplementation scenarios among the population under 2 years old in the 4 countries. We chose a dynamic mathematical model to capture the complex effects of maternal BMI and different supplementation scenarios on child development in the first 2 years of life. The model components included in the simulation and the relationships between them were planned a priori as informed by evidence from the GBD study and other literature sources. We modeled the effect of maternal BMI on birth weight and the effect of supplementation on birth weight, stunting, and wasting. We did not include the effect of supplementation on maternal risk factors. In our model, the effect of LBW and short gestation acts directly on mortality in the neonatal period. The effect of stunting and wasting acts on mortality and morbidity through protein energy malnutrition (for wasting only), measles, diarrheal diseases, and lower respiratory tract infections. These risk–outcome relationships were selected according to the GBD 2017 study risk–outcome pair criteria for convincing or probable evidence of causation [28] (**Fig 1**). Model implementation strategy was adapted and iteratively updated when model verification criteria were not met throughout model development. The iterative workflow process followed throughout model development is described in S1 Supplement. Parameter values for the intervention costs, the association between maternal BMI and birth weight, the hypothesized effect size of BEP for undernourished mothers in a best-case scenario, and the sensitivity analysis were revised after peer review, and the model was rerun.

For each country, we simulated a population of 100,000 live newborns with a model run length of 2 years. In accordance with the 2017 GBD study, birth weight exposure affects health outcomes during the neonatal period (0 to 27 days), and wasting and stunting exposures affect health outcomes from the neonatal period until 5 years of life. We chose a 2-year time horizon to capture the effect of in utero maternal supplementation exposure on child health outcomes in early life. We used fixed time increments of 1 day as the time step. We obtained 95% uncertainty intervals (UIs) to represent parameter and stochastic uncertainty across 100 iterations of model runs.

At model initialization, we assigned attributes to simulants including age (0 at birth), sex, maternal undernourishment status, supplementation coverage, risk exposures (birth weight as well as wasting and stunting z-scores), and possession of any congenital disease or conditions. We used location-, age-, and sex-specific GBD 2017 national average estimates as inputs for the population sex structure, risk exposure, and disease and mortality model components [28–31]. Details of maternal undernourishment status, coverage, and risk exposures are described below in the baseline model section. The attributes of each individual at the start of the simulation determined disease states, mortality, and morbidity at subsequent time steps using Monte Carlo sampling from model parameters. RRs, the population attributable fraction (PAF) between risk–outcome pairs, and population incidence rates determined probability of disease or condition (for detailed methodology of RR and PAF calculations, see Supplementary Appendix 1 of GBD 2017 Risk Factor Study [28]). Briefly, the RRs for a risk–outcome pair represent the risk of disease for a given exposure level relative to the theoretical minimum risk exposure level (TMREL;RR = 1 for TMREL).

            Incidence rate among exposure category = Population incidence*(1-PAF)*RR

            Incidence rate among TMREL = Population incidence*(1-PAF)

            Probability of disease or condition within time step = 1−*e*^(−incidence rate*time_step)^

A simulant’s cause state (x_i_, where x_1_ = 1 represents with disease or condition, and x_0_ = 0 represents without disease or condition), the all-cause mortality rate (ACMR), as well as cause-specific mortality rates (CSMRs), and excess mortality rates (EMRs) for modeled disease or conditions were used to determine the simulant’s mortality probability. The time of a simulant’s death in relation to the theoretical maximum life expectancy at that age determines years of life lost (YLLs).

            Mortality hazard = $\mathrm{ACMR}+\sum_{cause=x}^{n}\left(-CSMR+EMR(x_{i})\right)$, where x = disease or conditions in **Fig 1**.

            Probability of mortality within time step = 1−*e*^(−mortality hazard*time_step)^

Alive simulants may recover from a disease or condition according to the cause-specific remission rate. Disability weights associated with the disease or condition determined simulants’ years lived with disability (YLDs) [30]. We calculated DALYs by summing YLLs and YLDs.

We compared DALYs from different intervention scenarios with the DALYs from the scenario with baseline coverage of IFA supplementation. For intervention scenarios, we scaled up supplementation coverage to 90% where routine antenatal care (ANC) attendees receive (1) universal MMS; (2) universal BEP; or (3) MMS + targeted BEP (attendees with low prepregnancy BMI (<18.5 kg/m^2^) receive BEP containing MMS, while those above (BMI >18.5 kg/m^2^) receive MMS). We applied common random numbers between scenarios to ensure that differences in outcomes were due to the different interventions rather than stochastic variation. Data and software are held at https://github.com/ihmeuw/vivarium_gates_bep.

### Baseline model

#### Maternal undernourishment

Maternal undernourishment is a risk factor for LBW [7]. We stratified children into those born to (1) undernourished mothers (BMI <18.5 kg/m^2^); and (2) adequately nourished mothers (BMI ≥18.5 kg/m^2^). We obtained the prevalence of maternal undernourishment from the Demographic and Health Survey (DHS) and ongoing BEP trials where DHS data were unavailable (**Table 1**).

**Table 1:** Baseline coverage of ANC from skilled provider, coverage of IFA, and proportion undernourished by country with 95% CIs.

Country	ANC from skilled provider^†^ (%)	Women who took antenatal iron for 90+ days^‡^ (%)	Women who are undernourished according to BMI (<18.5 kg/m^2^) (%)
India	88.2 (87.4 to 89.0)	38.7 (31.0 to 46.4)	16.8 (13.4 to 20.2)^¶^
Pakistan	84.4 (80.5 to 87.8)	29.4 (23.5 to 35.3)	10.7 (8.6 to 12.8)^¶^
Mali	83.5 (79.1 to 87.5)	28.0 (22.4 to 33.6)	10.3 (8.2 to 12.4)^μ^
Tanzania	98.3 (97.7 to 98.8)	21.4 (17.1 to 25.7)	9.5 (7.6 to 11.4)^μ^

Note: Where 95% confidence ranges were unavailable, we applied a range of ±20% from the reported mean value as a plausible coefficient of variability.

^†^GBD 2017: Proportion of pregnant women receiving any ANC from a skilled provider.

^‡^Most recent available DHS: percentage of women with a live birth in the 5 (or 3) years preceding the survey who took 90+ days of iron tablets or syrup during ANC.

^μ^Most recent available DHS, among women aged 15 to 49.

^¶^Ongoing Gates trials, among pregnant women.

ANC, antenatal care; BMI, body mass index; CI, confidence interval; DHS, Demographic and Health Survey; GBD, Global Burden of Disease; IFA, iron and folic acid.

We assumed a crude birth weight difference of 138.46 g with a 95% confidence interval (CI) of 174.68 to 102.25 between maternal BMI strata, with higher birth weight babies born to mothers with BMI >18.5 kg/m^2^. We obtained this effect difference by conducting a literature search to identify birth weight effect sizes and pooling across 18 studies using a random effects model (S2 Supplement).

#### IFA baseline coverage

We obtained baseline IFA coverage from the latest available DHS (**Table 1**). We stratified the population into those who received IFA and those who did not. We assumed that infants born to IFA supplemented mothers had an average birth weight of +57.73g (95% CI: 7.66 to 107.79) greater than infants born to mothers not supplemented with IFA [32]. We assumed all women who were supplemented during pregnancy received supplements through ANC visits. We used the coverage proportion for pregnant women who received ANC from a skilled provider at least one time from GBD 2017 as the proportion of women who attend ANC visits and are thus able to receive supplementation.

We therefore have 4 subgroups of birth weights broken out from the GBD 2017 population mean birth weight such that the 4 groups’ (2 IFA strata by 2 BMI strata) weighted average equals the population mean. The difference in birth weight between groups reflects the mean effect differences we obtained from the literature.

#### Birth weight and child growth

In GBD 2017, the birth weight and gestational age exposure was modeled as an ordered polytomous joint distribution specifying the prevalence of births in 500 g by 2-week birth weight–gestational age categories [28]. We first converted this discrete exposure distribution into a continuous joint exposure distribution of birth weight and gestational age by assuming a uniform distribution of birth weights and gestational ages within each category. We assigned each simulant a continuously distributed birth weight and gestational age, which was mapped back to the appropriate risk category in GBD to obtain the RRs for all-cause mortality for the early neonatal (0 to 6 days) and late neonatal (7 to 27 days) age groups. The RRs used in this analysis were specific to each 500 g by 2-week birth weight–gestational age category such that the difference in mortality risk between 2 categories with different birth weights and the same gestational age varied across gestational age categories. Additionally, the difference in mortality risk from one birth weight category to the next with constant gestational age varied such that the magnitude of mortality risk reduction tended to be greater among the lower end of the birth weight distribution.

We used a propensity model for wasting and stunting. Each simulant was initialized with a “propensity” for wasting and stunting, and the simulant’s z-scores were determined by comparing this propensity to the overall age-specific z-score exposure prevalence in the population such that individual simulant’s wasting and stunting z-scores may change as they age, but their population percentile remains constant. The TMREL for wasting and stunting were defined as z-scores greater than −1 SD of WHO 2006 standard weight-for-length and length-for-age curves, respectively.

We used a correlation Spearman’s rho of 0.394 (95% UI: 0.353 to 0.433; SD: 0.020) for birth weight and length-for-age z-score (LAZ) at 6 months and Spearman’s rho of 0.308 (95% CI: 0.263 to 0.351; SD: 0.022) for birth weight and weight-for-length z-score (WLZ) at 6 months. The correlation coefficients were obtained from a pooled analysis of multicountry birth cohorts from the Etiology, Risk Factors, and Interactions of Enteric Infections and Malnutrition and the Consequences for Child Health (MAL-ED) study [33].

### Intervention model

**Fig 2:**
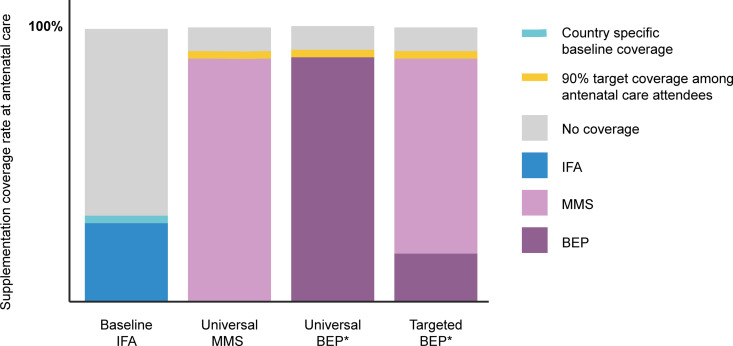
Visual diagram of supplementation coverage among women who attend ANC. Baseline IFA: scenario with country-specific reported coverage of IFA consumption; Universal MMS: scenario where 90% of antenatal attendees receive MMS; Universal BEP: scenario where 90% of antenatal attendees receive BEP; MMS + targeted BEP: scenario where 90% of antenatal attendees receive BEP if undernourished and receive MMS if adequately nourished according to prepregnancy BMI. *We modeled BEP scenarios twice: once with effects supported by ce and second with he suggested by some evidence. ANC, antenatal care; BEP, balanced energy protein (contains MMS); BMI, body mass index; ce, current evidence; he, hypothesized evidence; IFA, iron and folic acid; MMS, multiple micronutrient supplementation (contains IFA).

We modeled 3 intervention scenarios where 90% of ANC attendees received (1) universal MMS; (2) universal BEP; or (3) MMS + targeted BEP (**Fig 2**). We assume the formulation of MMS contains IFA and that BEP contains MMS, which contains IFA. In each scenario, IFA, MMS, and BEP supplementations were modeled to improve infant birth weight as supported by current evidence (ce) [9,15,32]. For BEP, we modeled a differential effect of BEP on birth weight by maternal nourishment status because there is some evidence to suggest that BEP may have a greater impact on birth weight among women with low prepregnancy body weight [15].

For interventions involving BEP, we modeled 2 additional scenarios with BEP hypothesized effects (he) on WLZ and LAZ scores at 6 months as suggested in Kusin and colleagues [19] and greater effect sizes for BEP on birth weight for undernourished women as suggested by some studies as a best-case scenario [16–18] (**Table 2**).

**Table 2:** Effects of different maternal intervention on birth weight and 6-month child growth indicators.

Supplementation	Infant outcomes	Comparison groups	Mean difference (95% CI)	Cost per beneficiary in 2021 USD (95% CI)^†^
IFA	Birth weight (g)	Unsupplemented	+57.73 g (7.66 to 107.79) (32)	17.37 (13.90 to 20.84) (13)
Multiple micronutrient	Birth weight (g)	Iron with or without folic acid	+45.16 g (32.31 to 58.02) (9)	19.47 (15.58 to 23.36) (13)
BEP (ce)	Birth weight (g)	Control or no intervention*	*Undernourished*: +66.96 g (13.13 to 120.78) (15)	India: 47.88 (38.30 to 57.46) (34) Pakistan: 45.96 (36.77 to 55.15) (34) Mali: 41.20 (32.96 to 49.44) (34) Tanzania: 41.90 (33.52 to 50.28) (34)
			*Adequately nourished*: +15.93 g (−20.83 to 52.69) (15)	
BEP (he^π^)	Birth weight (g)	Control or no intervention*	*Undernourished*: +136 g (79 to 193)** (16)	
			*Adequately nourished*: +15.93 g (−20.83 to 52.69) (15)	
BEP (he^π^)	LAZ score at 6 months	Unsupplemented	+0.3 (±0.1) (19)	
BEP (he^π^)	WLZ score at 6 months	Unsupplemented	+0.3 (±0.1) (19)	

Note: In each scenario, we applied effect sizes additively according to nutrients received.

*The trials from the 2015 Cochrane review compared a range of comparison groups. We interpreted the effect size as that of balanced energy plus vitamins and minerals versus vitamins and minerals.

**CI calculated from reported standard error as ±1.96 × SE.

πWe modeled 2 additional scenarios for BEP: once with effects supported by ce and second with he suggested by some evidence.

BEP, balanced energy protein; ce, current evidence; CI, confidence interval; he, hypothesized evidence; IFA, iron and folic acid; LAZ, length-for-age z-score; WLZ, weight-for-length z-score.

### Sensitivity analysis

We performed a sensitivity analysis around supplementation coverage in which we modeled the impact of each intervention scenario with coverage levels equal to the location-specific proportion of women who received IFA supplementation at baseline (**Table 1**).

### Data sources

#### Inputs parameterized to the GBD study

We used 1,000 draw-level location/age/sex-specific risk exposure distributions, disease prevalence at birth, disease incidence rate, CSMR, ACMR, and EMR from the GBD 2017 study to parameterize the population-level rates in our model. We assigned individual simulant-level measures in the baseline scenario according to coverage of IFA supplementation and maternal underweight such that (1) the birth weight and downstream outcome differences (**Fig 1**) among IFA-supplemented and unsupplemented individuals as well as undernourished and adequately nourished mothers reflected the effect sizes of IFA and maternal undernourishment; and (2) the population-level measures in our model reflected the GBD 2017 estimates.

#### Effect sizes and costs from published literature

**Table 2** presents the interventions, affected outcomes, effect sizes, and cost per supplementation course. We assumed a normal distribution of uncertainty for effect sizes. For the effect of MMS on birth weight, we reviewed studies from Keats and colleagues’ Cochrane review to obtain birth weight mean differences between MMS supplemented groups and iron with or without folic acid supplemented groups [9]. A total of 13 out of 18 trials reported mean birth weight shifts. We pooled the birth weight differences using a random effects model (S3 Supplement). For each scenario, we applied effect sizes additively according to supplements received.

For our cost analysis, we obtained the IFA and MMS costs from Kashi and colleagues [13]. These estimates considered costs of supplements, service delivery, and program expenses. We obtained costs for BEP supplementation from Scott and colleagues [34]. The authors calculated country-specific BEP costs by scaling the range of costs from Shekar and colleagues [35]. All costs reflect 180 days (6 months) of supplementation during pregnancy. The costs reflect a payer’s perspective as results from this study are intended to aid government or donor decision-making. Where there was an absence of quantified UIs from available sources, we assumed a plausible coefficient of variation of 20% about the point estimates. We did not include discounting as the ce on the benefits of supplementation occur mostly in the neonatal period, within 1 year of the supplementation period. We use half of GDP per capita as the cost-effective threshold for acceptable cost per DALY averted [22]. All costs were adjusted to August 2021 USD using the United States Bureau of Labor Statistics Consumer Price Index Inflation Calculator (accessed September 2021) [36].

### DALYs and ICERs

We summed YLLS and YLDs to obtain DALYs per 100,000 births for each scenario. We then compared each scenario’s DALYs to the reference baseline scenario’s DALYs to calculate averted DALYs. We tracked counts of antenatal supplement courses administered to calculate the total cost of the intervention for each scenario. We calculated the incremental cost-effectiveness ratios (ICERs) by dividing the difference in costs between 2 interventions (cost difference) by the difference in their benefits (DALYs averted).

### Model verification

We verified the results of our model, in particular the ACMRs, CSMRs, disease state transition rates, and risk exposure distributions against the estimates from GBD 2017 to confirm that the model estimates matched. We also verified the model’s maternal undernourishment and supplementation effect sizes with the input data.

This study is reported as per the Consolidated Health Economic and Evaluation Reporting Standards (CHEERS) Statement (S4 Supplement).

## Results

### Model verification

We compared the output parameters from our baseline model to the GBD 2017 input estimates (S5 Supplement). All model outputs were within 10% of GBD estimates used as model inputs, namely ACMRs, CSMRs, cause incidence, prevalence, and remission. Effect sizes between maternal nourishment strata and supplementation strata also matched our input data.

### Characteristics of baseline population

**Table 3** presents the mean birth weight, prevalence of LBW, prevalence of wasting and stunting, and their mean z-scores in the baseline population of our simulation. **Table 4** presents the baseline percentage of DALYs attributable to LBW, stunting, and wasting in our simulation. Tanzania had substantially higher mean birth weight and mean WLZ-scores than the other countries at baseline.

**Table 3:** Characteristics of baseline simulated populations of the 4 modeled countries with 95% CIs.

	India	Pakistan	Mali	Tanzania
Birth weight				
Mean birth weight, grams				
Overall	2,889 (2,880 to 2,899)	2,843 (2,818 to 2,876)	2,930 (2,899 to 2,993)	3,268 (3,190 to 3,320)
Maternal undernourished	2,771 (2,742 to 2,805)	2,718 (2,666 to 2,763)	2,803 (2,762 to 2,869)	3,140 (3,062 to 3,211)
Maternal adequately nourished	2,912 (2,903 to 2,926)	2,858 (2,834 to 2,891)	2,944 (2,909 to 3,010)	3,282 (3,200 to 3,336)
Prevalence of LBW	21.5% (20.8% to 22.1%)	23.6% (21.6% to 25.5%)	18.0% (15.5% to 19.7%)	7.7% (6.3% to 9.9%)
Wasting				
Mean WLZ at 6 m	−0.428 (−0.456 to −0.401)	−0.460 (−0.604 to −0.347)	−0.408 (−0.541 to −0.301)	−0.090 (−0.156 to −0.044)
% Not wasted	63.8% (63.4% to 64.2%)	67.8% (63%.7 to 71.0%)	67.9% (64.8% to 70.6%)	77.4% (75.5% to 78.8%)
% Mildly wasted (WLZ < −1)	19.6% (19.3% to 20.0%)	21.8% (19.4% to 25.1%)	20.3% (18.3% to 23.7%)	15.8% (15.1% to 16.7%)
% Moderately wasted (WLZ < −2)	10.6% (10.3% to 10.8%)	7.9% (6.2% to 9.5%)	8.4% (7.4% to 9.7%)	5.2% (4.6% to 5.9%)
% Severely wasted (WLZ < −3)	6.0% (5.8% to 6.3%)	2.6% (1.3% to 4.5%)	3.3% (2.1% to 4.7%)	1.6% (1.1% to 2.0%)
Stunting				
Mean LAZ at 6 m	−0.887 (−0.935 to −0·829)	−1.388 (−2.015 to −0.819)	−0.853 (−1.370 to −0.446)	−1.081 (−1.495 to −0.777)
% Not stunted	51.0% (50.1% to 52.1%)	36.4% (16.1% to 50.6%)	52.8% (30.2% to 66.0%)	45.6% (27.6% to 54.5%)
% Mildly stunted (LAZ < −1)	20.4% (19.6% to 21.4%)	26.1% (15.7% to 45.8%)	34.2% (21.2% to 55.9%)	30.2% (21.7% to 48.2%)
% Moderately stunted (LAZ < −2)	15.0% (14.6% to 15.5%)	20.7% (13.6% to 32.9%)	10.6% (7.6% to 25.5%)	17.0% (14.0% to 24.0%)
% Severely stunted (LAZ < −3)	13.6% (12.9% to 14.4%)	16.8% (8.0% to 30.4%)	2.4% (0.5% to 4.7%)	7.8% (4.1% to 10.7%)

Note: RRs for GBD risk–outcome pairs are above 1 for z-scores −1 to −3.

CI, confidence interval; GBD, Global Burden of Disease; LAZ, length-for-age z-score; LBW, low birth weight defined as <2,500 g; m, months; RR, relative risk; WLZ, weight-for-length z-score.

**Table 4:** Baseline DALYs attributable to modifiable risk factors among male and female children of under 2 years of age, 2017.

	India			Pakistan			Mali			Tanzania		
Cause attributable to	% of DALYs	Risk attribution	DALYs	% of DALYs	Risk attribution	DALYs	% of DALYs	Risk attribution	DALYs	% of DALYs	Risk attribution	DALYs
LBW												
All causes* affected	20.42%	49.27%	10.06%	39.01%	54.16%	21.13%	37.48%	45.17%	16.93%	30.38%	40.88%	12.42%
Total DALYs attributable			**10.06%**			**21.13%**			**16.93%**			**12.42%**
**Stunting**												
Measles	0.30%	21.23%	0.06%	0.17%	19.54%	0.03%	0.95%	15.79%	0.15%	0.69%	20.72%	0.14%
Diarrhea	4.71%	3.80%	0.18%	5.47%	9.72%	0.53%	6.30%	6.74%	0.42%	3.98%	5.35%	0.21%
Lower respiratory tract infection	4.26%	7.06%	0.30%	5.69%	11.33%	0.64%	8.12%	10.58%	0.86%	9.06%	11.67%	1.06%
Total DALYs attributable			**0.54%**			**1.21%**			**1.43%**			**1.41%**
**Wasting**												
Measles	0.30%	41.92%	0.13%	0.17%	33.86%	0.06%	0.95%	35.26%	0.33%	0.69%	22.13%	0.15%
Diarrhea	4.71%	21.33%	1.00%	5.47%	51.12%	2.80%	6.30%	52.22%	3.29%	3.98%	26.24%	1.04%
Lower respiratory tract infection	4.26%	31.31%	1.33%	5.69%	46.87%	2.67%	8.12%	62.04%	5.04%	9.06%	43.51%	3.94%
Protein energy malnutrition	0.57%	100.00%	0.57%	1.00%	100.00%	1.00%	9.00%	100.00%	9.00%	2.23%	100.00%	2.23%
Total DALYs attributable			**3.03%**			**6.52%**			**17.66%**			**7.37%**

*Causes of morbidity and mortality affected by LBW according to the 2017 GBD study criteria for causality include neonatal preterm birth, neonatal encephalopathy, lower respiratory tract infection, other neonatal disorders, neonatal sepsis, hemolytic disease and jaundice, upper respiratory tract infections, otis media, diarrhea, meningitis, and encephalitis.

DALY, disability-adjusted life year; GBD, Global Burden of Disease; LBW, low birth weight.

### DALYs, ICERs, and cost-effectiveness thresholds

**Fig 3:**
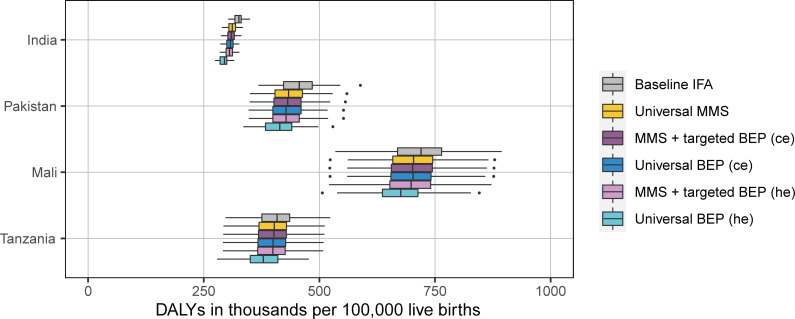
DALYs due to all causes among children under 2 in thousands per 100,000 live births in each simulated supplementation scenario by location. The width of the colored boxes represents the interquartile range (25th to 75th percentiles); the solid line in the box represents the median (50th percentile); the whiskers on either end of the box represent the minimum and maximum values; and the points represent outlier values. BEP, balanced energy protein; ce, scenarios with current evidence effects for BEP; DALY, disability-adjusted life year; he, scenarios with hypothesized effects for BEP; IFA, iron and folic acid; MMS, multiple micronutrient supplementation.

**Fig 4:**
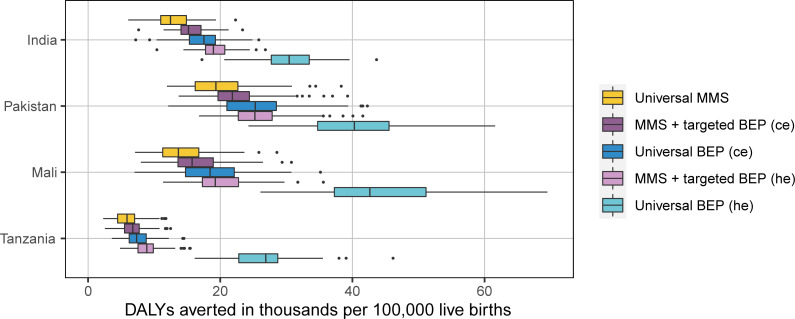
DALYs averted in thousands relative to the baseline scenario for each simulated supplementation intervention scenario among children under 2 per 100,000 live births by location. The width of the colored boxes represents the interquartile range (25th to 75th percentiles); the solid line in the box represents the median (50th percentile); the whiskers on either end of the box represent the minimum and maximum values; and the points represent outlier values. BEP, balanced energy protein; ce, scenarios with current evidence effects for BEP; DALY, disability-adjusted life year; he, scenarios with hypothesized effects for BEP; MMS, multiple micronutrient supplementation.

Our model yielded baseline burdens of 324,000 (95% UI: 307,000 to 343,000), 456,000 (95% UI: 376,000 to 543,000), 723,000 (95% UI: 600,000 to 872,000), and 406,000 (95% UI: 322,000 to 486,000) DALYs per 100,000 live births for India, Pakistan, Mali, and Tanzania, respectively (**Fig 3**). Assuming the proportion of women attending ANC remains unchanged and 90% of ANC attendees receive supplementation in our intervention scenarios, universal BEP averts the most DALYs followed by MMS + targeted BEP and then universal MMS compared to baseline IFA (**Fig 4**). Scenarios using the hypothesized effects for BEP resulted in more DALYs averted relative to baseline than the scenarios using the ce (**Fig 4**). The intervention effects per 100,000 live births are strongest in Pakistan, followed by Mali, India, and Tanzania. This follows the pattern in LBW prevalence and percentage of baseline DALYs attributable to LBW (**Table 4**).

**Fig 5:**
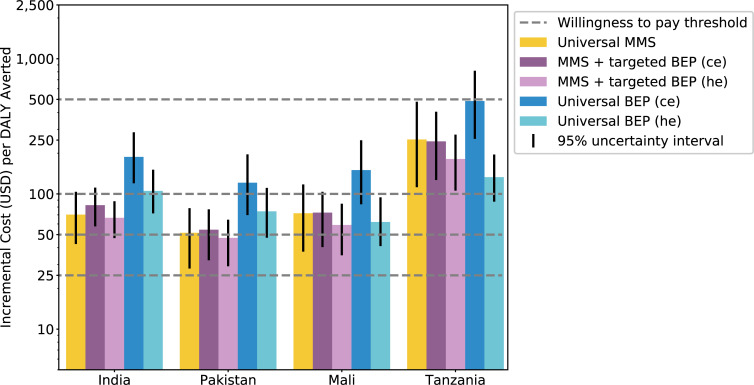
Incremental cost per DALY averted among the first 2 years of life in each modeled location for each simulated scenario relative to the baseline scenario (log-scale). BEP, balanced energy protein; ce, scenarios with current evidence effects for BEP; DALY, disability-adjusted life year; he, scenarios with hypothesized effects for BEP; MMS, multiple micronutrient supplementation.

The ICERs for universal MMS compared to baseline IFA were lowest for Pakistan, followed by India, Mali, and Tanzania at $52 (95% UI: 28, 78), $70 (95% UI: 43, 104), $72 (95% UI: 37, 118), and $253 (95% UI: 112, 481), respectively. The ICERs for MMS + targeted BEP relative to baseline IFA using ce effects for BEP were similar to those of universal MMS for each respective location at $54 (95UI: 32, 77), $83 (95% UI: 58, 111), $73 (95% UI: 40, 104), and $245 (95% UI: 127, 405). Universal BEP using ce for BEP effects was less cost-effective than universal MMS and MMS + targeted BEP scenarios (**Table 5, Fig 5**). For both the MMS + targeted BEP and universal BEP, ICERs using the hypothesized effects of BEP were lower than those using the ce for the effects of BEP. For Mali and Tanzania, universal BEP relative to baseline IFA using hypothesized effects for BEP had lower ICERs than universal MMS at $62 (95% UI: 41, 94) and $133 (95% UI: 88, 195), respectively. For each modeled location, universal MMS and MMS + targeted BEP ICERS were less than half of GDP (**Table 6**).

**Table 5:** Population-level effect and cost-effectiveness of interventions over first 2 years of life with 95% UIs.

	DALYs averted relative to baseline per 100,000 live births	Number treated per 100,000 live births	Total cost (2021 USD) per 100,000 live births	ICER (2021 USD per DALY averted) relative to baseline
**India**				
Baseline IFA	-	38,829 (25,830 to 42,930)	676,981 (536,418 to 830,096)	-
Universal MMS	13,014 (9,220 to 18,735)	79,402 (78,593 to 80,294)	1,551,238 (1,200,889 to 1,855,065)	70 (43 to 104)
Universal BEP (ce)	17,396 (11,035 to 23,875)	79,402 (78,593 to 80,294)	3,814,756 (2,953,189 to 4,561,916)	188 (120 to 285)
MMS + targeted BEP (ce)	15,584 (11,767 to 20,883)	MMS: 65,960 (64,540 to 67,603), BEP: 13,442 (12,247 to 14,758)	1,934,423 (1,487,349 to 2,322,707)	83 (58 to 111)
Universal BEP (he)	30,504 (21,526 to 38,574)	79,402 (78,593 to 80,294)	3,814,756 (2,953,189 to 4,561,916)	105 (72 to 151)
MMS + targeted BEP (he)	19,186 (15,064 to 24,467)	MMS: 65,960 (64,540 to 67,603), BEP: 13,442 (12,247 to 14,758)	1,934,423 (1,487,349 to 2,322,707)	67 (47 to 88)
**Pakistan**				
Baseline IFA	-	29,484 (27,216 to 32,572)	514,079 (406,470 to 631,709)	-
Universal MMS	19,931 (12,385 to 32,270)	75,852 (72,101 to 79,896)	1,481,906 (1,142,619 to 1,789,513)	52 (28 to 78)
Universal BEP (ce)	25,441 (14,267 to 40,379)	75,852 (72,101 to 79,896)	3,426,122 (2,642,158 to 4,030,132)	121 (70 to 196)
MMS + targeted BEP (ce)	22,541 (14,796 to 34,629)	MMS: 67,674 (64,299 to 71,418), BEP: 8,178 (7,445 to 9,033)	1,691,688 (1,351,421 to 2,002,914)	54 (32 to 77)
Universal BEP (he)	40,739 (26,320 to 58,666)	75,852 (72,101 to 79,896)	3,426,122 (2,642,158 to 4,030,132)	74 (47 to 111)
MMS + targeted BEP (he)	25,710 (17,848 to 37,635)	MMS: 67,674 (64,299 to 71,418), BEP: 8,178 (7,445 to 9,033)	1,691,688 (1,351,421 to 2,002,914)	47 (29 to 64)
**Mali**				
Baseline IFA	-	28,074 (25,903 to 30,983)	489,512 (387,484 to 601,238)	-
Universal MMS	14,658 (8,691 to 24,800)	75,044 (70,709 to 79,672)	1,466,118 (1,129,808 to 1,774,937)	72 (37 to 118)
Universal BEP (ce)	18,683 (11,059 to 30,763)	75,044 (70,709 to 79,672)	3,080,566 (2,568,366 to 3,720,033)	150 (84 to 249)
MMS + targeted BEP (ce)	16,608 (11,308 to 26,125)	MMS: 67,254 (63,410 to 71,465), BEP: 7,790 (7,084 to 8,658)	1,633,605 (1,355,894 to 1,945,998)	73 (40 to 104)
Universal BEP (he)	43,657 (28,017 to 61,122)	75,044 (70,709 to 79,672)	3,080,566 (2,568,366 to 3,720,033)	62 (41 to 94)
MMS + targeted BEP (he)	20,233 (13,885 to 30,779)	MMS: 67,254 (63,410 to 71,465), BEP: 7,790 (7,084 to 8,658)	1,633,605 (1,355,894 to 1,945,998)	59 (35 to 85)
**Tanzania**				
Baseline IFA	-	21,463 (19,789 to 23,713)	374,242 (296,461 to 460,573)	-
Universal MMS	6,010 (2,891 to 11,347)	88,464 (87,880 to 89,111)	1,728,290 (1,339,156 to 2,069,445)	253 (112 to 481)
Universal BEP (ce)	7,576 (4,079 to 12,215)	88,464 (87,880 to 89,111)	3,749,575 (3,104,093 to 4,530,882)	487 (256 to 814)
MMS + targeted BEP (ce)	6,843 (3,775 to 11,824)	MMS: 79,992 (79,045 to 81,055), BEP: 8,472 (7,728 to 9,256)	1,921,643 (1,576,711 to 2,254,872)	245 (127 to 405)
Universal BEP (he)	26,347 (17,587 to 36,812)	88,464 (87,880 to 89,111)	3,749,575 (3,104,093 to 4,530,882)	133 (88 to 195)
MMS + targeted BEP (he)	8,991 (5,664 to 14,505)	MMS: 79,992 (79,045 to 81,055), BEP: 8,472 (7,728 to 9,256)	1,921,643 (1,576,711 to 2,254,872)	181 (106 to 274)

ICER is the difference in cost between 2 interventions, divided by the difference in their benefit.

BEP, balanced energy protein; ce, scenarios with current evidence effects for BEP; DALY, disability-adjusted life year; he, scenarios with hypothesized effects for BEP; ICER, incremental cost-effectiveness ratio; IFA, iron and folic acid; MMS, multiple micronutrient supplementation; UI, uncertainty interval.

**Table 6:** GDP per capita and cost-effectiveness thresholds in 2020 USD with simulated cost-effectiveness ratios in 2021 USD per DALY averted relative to baseline with 95% UIs.

Country	GDP per capita [37]	Half of GDP per capita	Incremental cost of universal MMS per DALY averted relative to baseline	Incremental cost of MMS + targeted BEP (ce) per DALY averted relative to baseline	Incremental cost of MMS + targeted BEP (he) per DALY averted relative to baseline
India	$2,108	$1,054	70 (43 to 104)	83 (58 to 111)	67 (47 to 88)
Pakistan	$1,285	$643	52 (28 to 78)	54 (32 to 77)	47 (29 to 64)
Mali	$891	$446	72 (37 to 118)	73 (40 to 104)	59 (35 to 85)
Tanzania	$1,122	$561	253 (112 to 481)	245 (127 to 405)	181 (106 to 274)

BEP, balanced energy protein; ce, current evidence effects for BEP; DALY, disability-adjusted life year; GDP, gross domestic product; he, hypothesized effects for BEP; UI, uncertainty interval.

**Fig 6:**
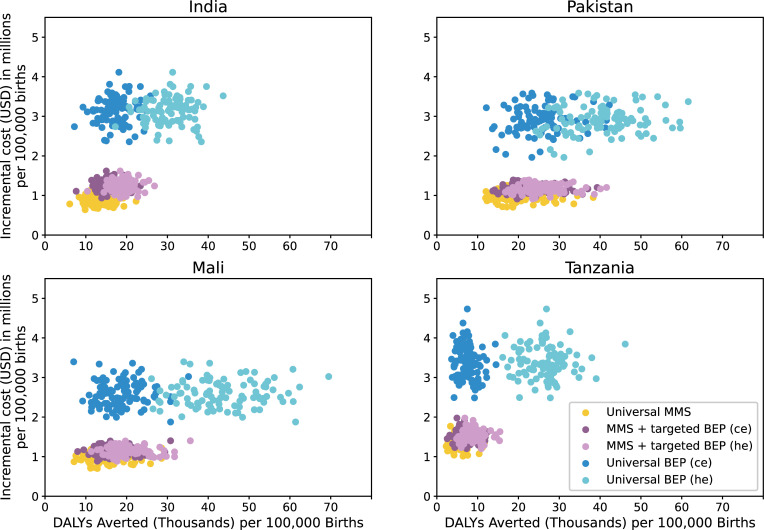
Incremental cost and DALYs averted among the first 2 years of life per 100,000 births for each simulated scenario relative to the baseline scenario in each modeled location. *Each point in this figure represents one of the 100 simulation runs performed, each of which used varying parameter values within the parameter UIs used in our model. BEP, balanced energy protein; ce, scenarios with current evidence effects for BEP; DALY, disability-adjusted life year; he, scenarios with hypothesized effects for BEP; MMS, multiple micronutrient supplementation; UI, uncertainty interval.

Offering BEP to all ANC attendees was least cost-effective, costing between $121 and $487 per DALY (mean estimates) for the modeled countries based on ce effect sizes (**Figs 5 and 6**). **Table 6** presents the DALYs averted, numbers treated, total cost of treatment, and ICERs per 100,000 live births for each scenario.

### Sensitivity analysis of supplementation coverage

When intervention coverage was implemented at the same level of baseline IFA coverage, fewer DALYs were averted per 100,000 births, and ICERs were lower in comparison to intervention coverage of 90% of ANC attendees. In this sensitivity analysis, the universal MMS scenario resulted in 3,746 (95% UI: 2,238, 5,846), 4,144 (95% UI: 2,284, 6,904), 2,950 (95% UI: 1,492, 4,796), and 662 (95% UI: 176, 1,364) DALYs averted relative to the baseline scenario per 100,000 live births for India, Pakistan, Mali, and Tanzania, respectively. Likewise, the MMS + targeted BEP scenario using ce effect sizes resulted in 4,974 (95% UI: 3,158, 7,033), 5,168 (95% UI: 2,854, 7,956), 3,685 (95% UI: 1,977, 5,674), and 864 (95% UI: 217, 1,669) DALYs averted for the respective locations.

The universal MMS intervention was more cost-effective in the sensitivity analysis than coverage for 90% of ANC attendees at $23 (95% UI: 13, 39), $16 (95% UI: 9, 26), $22 (95% UI: 12, 37), and $72 (95% UI: 34, 292) per DALY averted relative to baseline for India, Pakistan, Mali, and Tanzania, respectively. Likewise, the BEP interventions were slightly more cost-effective in the sensitivity analysis, with $56 (95% UI: 38, 81), $29 (95% UI: 18, 49), $35 (95% UI: 22, 62), and $135 (95% UI: 112, 415) per DALY averted relative to baseline in the respective locations in the MMS + targeted BEP scenario using ce effect sizes.

## Discussion

Inadequate maternal nutritional intake is a major contributor to poor birth outcomes [7]. To improve birth outcomes, WHO recommends antenatal supplementation with multiple micronutrients that include IFA in the context of rigorous research [8] and BEP in populations with over 20% burden of undernourished pregnant women [14]. As countries consider investing in MMS, there are opportunities to integrate supplement delivery with optimized formulations of BEP containing MMS, so that micronutrient and energy deficits can be met within a single vehicle. While some cost-effectiveness analyses have shown that switching from IFA to MMS is very cost-effective in some countries [10–13], there are no detailed studies, to the best of our knowledge, estimating the cost-effectiveness of BEP or comparing the cost-effectiveness of MMS and BEP [26]. Our study modeled the currently available evidence from Cochrane Systematic Reviews on antenatal IFA, MMS, and BEP supplementation as well as hypothesized effects of BEP as suggested by some evidence in a best-case scenario. We explored the impact of targeting undernourished women for BEP and found that MMS + targeted BEP averts more DALYs and remains cost-effective compared to universal MMS. Targeting may be an attractive and cost-effective strategy to consider, especially in countries that do not meet WHO’s 20% undernourishment prevalence. The strength of our approach came from using dynamic, individual-based microsimulation that leveraged draw-level estimates from the 2017 GBD study to compare the impact on DALYs and cost-effectiveness of different supplementation scenarios. Our study provides decision-makers a side-by-side comparison of the incremental cost-effectiveness of scaling strategies for MMS and BEP compared to current baseline levels of antenatal IFA consumption in 4 countries with a considerable burden of undernourishment among pregnant women.

Our ICERs for universal MMS were greater than a recent cost-effectiveness analysis of antenatal MMS supplementation, which estimated $22.47, $22.64, and $61.82 per DALY for India, Pakistan, and Tanzania, respectively (no data available for Mali) using the MMS Cost Benefit Tool [10]. However, the MMS Cost Benefit Tool only considered commodity costs which may have underestimated the costs. Both studies showed an approximate 3 times greater cost per DALY in Tanzania than in India and Pakistan. These results may reflect the differences in baseline characteristics of the populations: Since nutritional supplementations mainly affect birth weight, the greatest gains were made in countries with higher baseline prevalence of LBW and higher percentage of DALYs attributable to LBW. Tanzania has an overall higher mean birth weight at baseline, which means additional improvement in birth weight from supplementation may not have as great an impact on averting DALYs.

Targeting BEP for low BMI mothers and providing MMS to non-low BMI mothers averts more DALYs and is as cost-effective as universal MMS when using the currently available evidence for BEP effects. When considering the hypothesized best-case effects of BEP, MMS + targeted BEP averts more DALYs and is even more cost-effective than universal MMS. WHO has proposed using 1 to 3× GDP per capita as the cost-effective threshold in recommending adoption of new interventions; however, some have argued that this WHO threshold may still be too expensive and thresholds of half of GDP per capita are more reasonable [37]. Average GDP per capita (2020 USD) in 2019 for low-income countries was $780 while that for lower-middle income countries was $2180 [38]. Under these thresholds, targeted BEP scenarios were very cost-effective at under $100 per DALY for India, Pakistan, and Mali but over $100 threshold at $245 per DALY for Tanzania.

ICERs for universal BEP relative to baseline using the ce for BEP effects were consistently higher than ICERs for universal MMS relative to baseline. Horton and colleagues ranked the cost-effectiveness of 93 interventions for low- and middle-income countries and placed BEP as potentially appropriate for consideration in upper-middle countries at $500 per DALY [22]. Among modeled countries using ce effects, our estimates for BEP were under $500 per DALY, although the upper bound of the UI was greater than $500 for Tanzania. When considering the hypothesized effects of BEP on birth weight and its effects on child wasting and stunting in the best-case scenario, the ICERs for universal BEP relative to baseline were lower than the ICERs for universal MMS in Mali and Tanzania, but remained higher in India and Pakistan. This trend of greater relative cost-effectiveness of universal BEP under the hypothesized effects of BEP is consistent with the greater proportion of combined burden attributable to child stunting and wasting in our modeled locations. This suggests that if the hypothesized effects of BEP on child wasting and stunting were realized, it may be more cost-effective in locations with high wasting and stunting burdens. Still, increasing caloric intake through BEP for all women is expensive. Additionally, the prevalence of obesity and overweight is increasing in low- and middle-income countries; among our modeled countries, the prevalence of overweight and obese women ranged from 20% to 50% (S2 Supplement). Women with overweight or obesity are at higher risk of fetal overgrowth and macrosomia (≥4,000 g) [23], and the effect BEP supplementation among this group is still unknown.

Our study is limited by some key assumptions. First, the effect size of BEP on birth weight may be hard to generalize across different settings due to heterogeneity in the BEP product supplementation period and comparison groups used in the trials. Studies assessing the effect of BEP have used different supplement compositions such as chocolate-flavored liquid, biscuits, dry powder, and combination of foods such as milk, bread, oil, beans, and maize [15]. This makes it difficult to put a standard formula or price on BEP if standard formulas are not used. Cost and acceptability of the BEP supplements will depend on local preferences and availability. Different studies also had different supplementation durations, which may affect both intervention cost and impact. Additionally, studies varied in the comparison interventions in the control groups: some studies used alternative interventions such as vitamins and minerals only or low-energy protein source, placebo pills, or no interventions as the control group. Nonetheless, we assumed that the effect size reported in the literature is that of BEP compared to MMS.

Second, we applied our interventions to 90% of women who attend ANC, which may not be easy to achieve without substantial effort. The proportion of women who took antenatal iron for 90 or more days is low (20% to 40%) among our modeled countries. Multiple barriers that affect the effective coverage of IFA include pregnant women’s beliefs that hinder early ANC attendance, poor healthcare worker performance, verticalization of healthcare delivery, lack of availability of commodities, and irregular supply of essential pharmaceuticals [39]. Our sensitivity analysis estimated a lower population-level impact if intervention coverages only achieved the level of baseline IFA coverage. Additionally, timing of ANC attendance also varies and those who attend late in pregnancy would not receive maximum benefit. Extra costs may be needed to encourage women to attend ANC early to achieve effect sizes seen in the trials. Adherence to daily supplementation may also be lower in real-world conditions than observed in trials (although palatable and MMS-integrated BEP formulations might improve adherence). Additionally, our model does not consider differential utilization of ANC services by factors such as residency (urban versus rural) or maternal age, although such differences have been documented [40]. If supplementation interventions do not reach higher risk groups such as rural and/or young mothers, our model may overestimate their impact. Interventions will need to address local health system constraints and conduct robust implementation research to improve the uptake and adherence to supplementation programs.

Third, current available evidence may have underestimated the effect of BEP. The small improvement in birth weight associated with BEP may reflect the small net increment in energy intake achieved in the trials [15]. Substitution of the BEP supplement for the main diet, as well as nonadherence, has been postulated as possible explanations [15]. In the Gambia, high-energy groundnut biscuits provided to chronically undernourished women antenatally improved birth weight by much more: 136 g compared with those not supplemented antenatally [16]. The authors argued that supplementation is most effective when there is a large energy gap to fill [16,41]. While we attempted to address the greater magnitude of BEP’s impact among babies at the lower end of the birth weight distribution by stratifying birth weight by maternal undernourishment status, we may not have captured the full extent of the additional benefit that BEP may have for nutritionally vulnerable pregnancies. Further, the effect of maternal supplementation regimens has been shown to be greater among anemic mothers [42], which we did not consider in our simulation. Therefore, our modeled interventions may be more effective in countries with high maternal anemia burdens, which is not reflected in our results. Additionally, BEP’s impact on long-term physical growth has not been thoroughly examined [15,20]. One study from Indonesia found greater weight and length growth in children born to mothers who were given high-energy supplements compared to those born to mothers given a low-energy supplement [19]. More high-quality studies with long-term follow-up are needed to draw conclusions for the effect of BEP on birth weight and child growth. Finally, reviews of MMS and BEP do not show strong evidence for reduction in preterm births or an increase in average gestational age (for BEP) [9,15]. Hence, we assume the improvement in LBW (<2,500 g) reported in the literature is captured by improved birth weight independent of gestational age and we did not model any effect on gestational weeks. Notably, while we considered uncertainty in the parameters used in this model, which are reflected in the UIs of our results, we did not attempt to quantify structural uncertainty regarding the relationships between parameters nor heterogeneity at the subnational level in our model.

Fourth, our cost model accounts only for the supplementation period of 6 months for IFA, MMS, and BEP; we did not account for varying duration of supplementation, which may depend on timing of ANC visits if supplements were distributed through facilities. We only considered the costs to payers and did not consider other societal costs, such as time spent attending ANC for women. However, since ANC has multiple benefits, these costs may be shared among other facility-based antenatal interventions.

Finally, our model only estimated the effect of supplementation on outcomes among children under 2 and did not consider potential effects on maternal outcomes or outcomes among children that occur beyond the first 2 years of life. If we had included maternal outcomes as well, the interventions are likely to be more cost-effective.

Our study shows that MMS in combination with targeted BEP averts more DALYs than universal MMS compared to baseline IFA while remaining cost-effective (under $100 per DALY for India, Pakistan, and Mali and under $250 per DALY for Tanzania). As countries begin to consider using MMS in alignment with recently updated WHO guidelines, targeted BEP could be considered as an additional cost-effective strategy to maximize benefit and synergize program implementation.

## Supporting information

S1 Supplement Model development workflow. (DOCX)

S2 Supplement Meta-analysis of maternal BMI and infant birth weight. BMI, body mass index.
(DOCX)

S3 Supplement Meta-analysis of birth weight shift of multiple micronutrients versus iron with or without folic acid control. (DOCX)

S4 Supplement CHEERS checklist. CHEERS, Consolidated Health Economic and Evaluation Reporting Standards. (DOCX)

S5 Supplement Model verification plots comparing GBD study inputs and simulation outputs. GBD, Global Burden of Disease. (DOCX)

S6 Supplement Sensitivity analysis figures. (DOCX)

## Abbreviations

ACMR: all-cause mortality rate
ANC: antenatal care
BEP: balanced energy protein
BMI: body mass index
ce: current evidence
CHEERS: Consolidated Health Economic and Evaluation Reporting Standards
CI: confidence interval
CSMR: cause-specific mortality rate
DALY: disability-adjusted life year
DHS: Demographic and Health Survey
EMR: excess mortality rate
GBD: Global Burden of Disease
ICER: incremental cost-effectiveness ratio
IFA: iron and folic acid
IUGR: intrauterine growth restriction
LAZ: length-for-age z-score
LBW: low birth weight
MAL-ED: Etiology, Risk Factors, and Interactions of Enteric Infections and Malnutrition and the Consequences for Child Health
MMS: multiple micronutrient supplementation
PAF: population attributable fraction
RR: relative risk
SGA: small for gestational age
UI: uncertainty interval
WHO: World Health Organization
WLZ: weight-for-length z-score
YLDs: years lived with disability
YLLs: years of life lost
