# Supplementary material for: Cost-effectiveness of antenatal multiple micronutrients and balanced energy protein supplementation compared to iron and folic acid supplementation in India, Pakistan, Mali, and Tanzania: A dynamic microsimulation study
Source: PLoS Med. 2022 Feb 22;19(2):e1003902. doi: 10.1371/journal.pmed.1003902 (PMC8863292; doi:10.1371/journal.pmed.1003902)
Supplement: S1 Supplement — (DOCX) [file pmed.1003902.s001.docx]

**SUPPLEMENT 1**

**Model Development Workflow**

The team has developed an eight-phase model development workflow with phase-specific outputs.


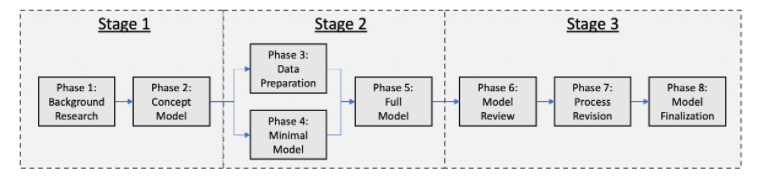


*Roles*

Researcher

The Researcher leads the model development process by guiding the background research, conceptualizing possible modeling strategies, validating those strategies with domain experts, guiding the conceptual development of software that will run the model, and generating analytics for model inputs and outputs. The researcher may develop tools in support of these processes.

Engineer

The Engineer produces all of the code to run the model, including the development of model components and observers, as well as the development of tools in support of model and input data analytics.

*Phase Descriptions*

Phase 1: Background Research

Complete a *Systematic Search* and generate a ROUGH first draft of an *Intervention Report.*

Phase 2: Concept Model Development

Complete a *Systematic Review* and a *Concept Model Document*.

Phase 3: Data Extraction and Minimal Modeling

Generate a *Data Artifact* and update the *Intervention Report* to include the current status.

Phase 4: Minimal Simulation Model Implementation

*This phase can progress simultaneously with Phase 3.*

Phase 5: Full Model Implementation

Add non-minimal components, validate results, and iteratively update until a final model is created.

Phase 6: Simulation Model Review

Results are presented to the full team for final validation.

Phase 7: Process Revision

The team discusses methods for iterative workflow improvement and brainstorms new tools to facilitate future model development.

Phase 8: Finalization

Models are archived and published.

Phase 9: Celebration

Pat self on back. Maybe get ice cream.
