# Supplementary material for: Cost-effectiveness of antenatal multiple micronutrients and balanced energy protein supplementation compared to iron and folic acid supplementation in India, Pakistan, Mali, and Tanzania: A dynamic microsimulation study
Source: PLoS Med. 2022 Feb 22;19(2):e1003902. doi: 10.1371/journal.pmed.1003902 (PMC8863292; doi:10.1371/journal.pmed.1003902)
Supplement: S2 Supplement — BMI, body mass index. (DOCX) [file pmed.1003902.s002.docx]

**SUPPLEMENT 2**

**Meta-analysis of maternal BMI and infant birthweight**

Our simulation relied on an estimation of the difference in infant birthweight between underweight mothers (pre-pregnancy body mass index (BMI) <18.5 kg/m^2^) and non-underweight mothers to model the effect between maternal nourishment status and infant birthweight. Our simulation necessitated a mean difference in grams rather than ratios that were readily available in existing meta-analyses.

To obtain an estimate of the needed quantity, we reviewed the systematic reviews of Yu et al. 2013 [1], Rahman et al. 2015 [2] and Liu et al. 2016 [3] for studies that reported mean birthweight by maternal nourishment category from low- or middle-income countries. We extracted the crude mean differences unadjusted for confounders because this value is used for the baseline adjustment (not used as a causal intervention effect). We conducted an additional literature search on Google Scholar and PubMed for studies using the search terms [“maternal underweight” OR “pre-pregnancy BMI”] AND “birth outcomes” OR “birthweight” from the year 2015 to 2021. We included 18 papers that reported mean birthweight differences between underweight women with pre-pregnancy or first trimester BMI below <18.5 kg/m^2^ and non-underweight women with BMI ≥18.5 kg/m^2^. If more than one ‘non-low’ birthweight groups were reported, we used the weighted average of the mean birthweight of all the non-low birthweight groups. We pooled the mean difference in birthweight using a random effects meta-analysis model in STATA 14 to obtain an effect of -138.4 (-102.3 to -174.5)**.** BMI groupings are according to WHO standards unless otherwise specified: underweight <18.5kg/m^2^; normal weight ≥18·5kg/m^2^ to <25kg/m^2^; overweight ≥18.5kg/m^2^ to <25kg/m^2^; obese ≥18.5kg/m^2^ to <25kg/m^2^. We assessed the studies risk of bias using the Newcastle-Ottawa Scale (NOS).

| **Table 1: Pre-pregnancy or first trimester body mass index (BMI) groupings and mean birthweight by study** | | | | | | | | |
| --- | --- | --- | --- | --- | --- | --- | --- | --- |
| **Study** | **Location** | **BMI groupings** | **N (%)** | **mean (SD)** | **Low BMI** | | **non-low BMI** | |
|  |  |  |  |  | **N** | **Mean (SD)** | **N** | **Mean (SD)** |
| Jeric 2013[4] | Split, Croatia | Underweight | 351 (7.5%) | 3343 (428) | 351 | 3343 (428) | 4327 | 3540 (113) |
|  |  | Normal weight | 3688 (78.8%) | 3511 (48) |  |  |  |  |
|  |  | Overweight | 550 (11.8%) | 3698 (492) |  |  |  |  |
|  |  | Obese | 89 (1.9%) | 3751 (509) |  |  |  |  |
| Sharifzadeh 2015 [5] | Tehran, Iran | Underweight | 21 (5.3%) | 2808 (648) | 21 | 2808 (648) | 375 | 3010 (639) |
|  |  | Normal weight | 198 (50%) | 2924 (594) |  |  |  |  |
|  |  | Overweight | 117 (29.5%) | 3026 (742) |  |  |  |  |
|  |  | Obese | 60 (15.2%) | 3265 (585) |  |  |  |  |
| Xiao 2017 [6] | Shanghai, China | Underweight | 120 (23.5%) | 3268 (368) | 120 | 3268 (336) | 390 | 3410 (462) |
|  |  | Normal weight^¶^ | 336 (65.9%) | 3390 (465) |  |  |  |  |
|  |  | Overweight and obese^¶^ | 54 (10.6%) | 3536 (448) |  |  |  |  |
| Soltani 2017 [7] | Western Sumatra, Indonesia | Underweight | 107 (20.1%) | 3140 (370) | 107 | 3140 (370) | 425 | 3177 (400) |
|  |  | Normal weight | 347 (65.2%) | 3159 (382) |  |  |  |  |
|  |  | Overweight | 72 (13.5%) | 3222 (479) |  |  |  |  |
|  |  | Obese | 6 (1.1%) | 3683 (519) |  |  |  |  |
| Gondwe 2018[8] | Semi-urban/ semi-rural area of southern Malawi | Underweight | 62 (5%) | 2939 (447) | 62 | 2939 (447) | 1074 | 2978 (446) |
|  |  | Normal weight | 941 (83%) | 2973 (447) |  |  |  |  |
|  |  | Overweight* | 133 (12%) | 3012 (440) |  |  |  |  |
| Nowak 2019[9] | Cracow, Poland | Underweight | 43 (9%) | 3197 (455) | 43 | 3197 (455) | 431 | 3317 (566) |
|  |  | Normal weight | 313 (66%) | 3294 (545) |  |  |  |  |
|  |  | Overweight | 91 (19%) | 3383 (581) |  |  |  |  |
|  |  | Obese | 27 (6%) | 3368 (760) |  |  |  |  |
| Bhowmik 2019[10] | Daka city, Bangledesh | Underweight | 38 (27.5%) | 2700 (400) | 38 | 2700 (400) | 100 | 2833 (400) |
|  |  | Normal weight^µ^ | 67 (48.6%) | 2800 (400) |  |  |  |  |
|  |  | Overweight^µ^ | 33 (23.9%) | 2900 (400) |  |  |  |  |
| Tang 2021[11] | China | Underweight | 136,287 (20%) | 3142 (136) | 136287 | 3142 (136) | 532814 | 3192 (387) |
|  |  | Normal | 448,439 (67%) | 3187 (385) |  |  |  |  |
|  |  | Overweight | 69,819 (10%) | 3214 (394) |  |  |  |  |
|  |  | Obese | 14,556 (2%) | 3224 (412) |  |  |  |  |
| Locks 2021[12] | India | Severely underweight | 457 (4%) | 2560 (512) | 5305 | 2625 (525) | 6192 | 2701 (540) |
|  |  | Underweight | 4848 (42%) | 2631 (526) |  |  |  |  |
|  |  | Normal | 5099 (44%) | 2686 (537) |  |  |  |  |
|  |  | Overweight | 1093 (10%) | 2769 (554) |  |  |  |  |
| Gul 2020[13] | Pakistan | Underweight | 110 (4%) | 2990 (680) | 110 | 2990 (680) | 2656 | 3176 (594) |
|  |  | Normal | 1290 (47%) | 3130 (620) |  |  |  |  |
|  |  | Overweight | 910 (33%) | 3210 (570) |  |  |  |  |
|  |  | Obese | 456 (16%) | 3240 (570) |  |  |  |  |
| Agbota 2020[14] | Benin | Underweight | 23 (9%) | 2835 (454) | 23 | 2835 (454) | 237 | 3048 (404) |
|  |  | Normal | 175 (67%) | 3030 (403) |  |  |  |  |
|  |  | Overweight | 43 (17%) | 3071 (391) |  |  |  |  |
|  |  | Obese | 19 (7%) | 3164 (438) |  |  |  |  |
| Mohammadi 2019[15] | Iran | Underweight | 198 (5%) | 3144 (448) | 198 | 3144 (448) | 4199 | 3218 (440) |
|  |  | Normal | 2293 (52%) | 3176 (447) |  |  |  |  |
|  |  | Overweight | 1434 (33%) | 3263 (423) |  |  |  |  |
|  |  | Obese | 472 (11%) | 3289 (455) |  |  |  |  |
| Pongcharoen 2016 [16] | Thailand | Underweight | 65 (17%) | 2988 (478) | 65 | 2988 (478) | 313 | 3135 (435) |
|  |  | Normal | 259 (69%) | 3116 (405) |  |  |  |  |
|  |  | Overweight | 54 (14%) | 3224 (579) |  |  |  |  |
| Ding 2016 [17] | China | Underweight | 2365 (23%) | 3303 (440) | 2365 | 3303 (440) | 7886 | 3435 (464) |
|  |  | Normal | 7240 (71%) | 3425 (459) |  |  |  |  |
|  |  | Overweight | 646 (6%) | 3552 (519) |  |  |  |  |
| Abubakari 2015 [18] | Ghana | Underweight | 16 (3.8%) | 2280 (420) | 16 | 2280 (420) | 403 | 2987 (538) |
|  |  | Normal | 242 (57.8%) | 2690 (430) |  |  |  |  |
|  |  | Overweight | 105 (25.1%) | 3260 (640) |  |  |  |  |
|  |  | Obese | 56 (13.4%) | 3760 (810) |  |  |  |  |
| Sahu 2007[19] | India | Lean | 46 (12.1%) | 2581 (482) | 46 | 2581 (482) | 334 | 2820 (545) |
|  |  | Normal | 205 (53.9%) | 2808 (479) |  |  |  |  |
|  |  | Overweight | 99 (26.1%) | 2835 (652) |  |  |  |  |
|  |  | Obese | 30 (8%) | 2855 (640) |  |  |  |  |
| Yekta 2006 [20] | Iran | Lean | 30 (11%) | 3103 (621) | 30 | 3103 (621) | 240 | 3298 (660) |
|  |  | Normal | 140 (52%) | 3256 (651) |  |  |  |  |
|  |  | Overweight | 52 (19%) | 3252 (650) |  |  |  |  |
|  |  | Obese | 48 (18%) | 3470 (694) |  |  |  |  |
| Ronnenberg 2003[21] | China | Severely underweight | 157 (27%) | 2966 (401) | 157 | 2966 (401) | 418 | 3180 (501) |
|  |  | Moderately underweight | 146 (25%) | 3159 (418) |  |  |  |  |
|  |  | Normal | 272 (47%) | 3192 (477) |  |  |  |  |
| *BMI ≥ 25.0 kg/m2  † SD approximated as ±20% of mean  ¶ women were divided into four groups based on pre-pregnancy BMI according to categories defined by the Working Group on Obesity in China as follows: underweight (BMI < 18.5 kg/m2), normal weight (18.5 kg/m2 ≤ BMI < 24.0 kg/m2), overweight (24.0 kg/m2 ≤ BMI < 28.0 kg/m2), and obese (BMI ≥ 28.0 kg/m2)  µ Normal weight (BMI 18.5 to < 23 kg/m2) and overweight (BMI ≥ 23 kg/m2) according to the Asian pre-pregnancy categories | | | | | | | | |

Table 2 shows the distribution of weight among women in the modeled countries. Maternal underweight ranges from 8.5% to 22.9% whereas maternal overweight and obese ranges from 20.7% to 52.2%. The distribution of weight in our model populations are comparable to those reported in the studies.

| **Table 2: Distribution of weight among women in India, Pakistan, Mali and Tanzania from most recent Demographic Health Survey** | | | | | | |
| --- | --- | --- | --- | --- | --- | --- |
| **Country** | **Survey** | **Underweight women with BMI (<18.5)** | **Normal weight women with BMI (18.5-24.9)** | **Overweight women with BMI (25.0-29.9)** | **Obese women with BMI (≥30.0)** | **Overweight and obese women with BMI (≥25.0)** |
| India | 2015-16 DHS | 22.9% | 56.4% | 15.5% | 5.1% | 20.7% |
| Pakistan | 2017-18 DHS | 8.5% | 39.3% | 30.4% | 21.8% | 52.2% |
| Mali | 2018 DHS | 10.3% | 61.9% | 19.0% | 8.7% | 27.8% |
| Tanzania | 2015-16 DHS | 9.5% | 62.1% | 18.4% | 10.0% | 28.4% |

| **Figure 1: Forest plot of the association between pre-pregnancy body mass index (BMI) and low birthweight (18 studies)** |
| --- |
| 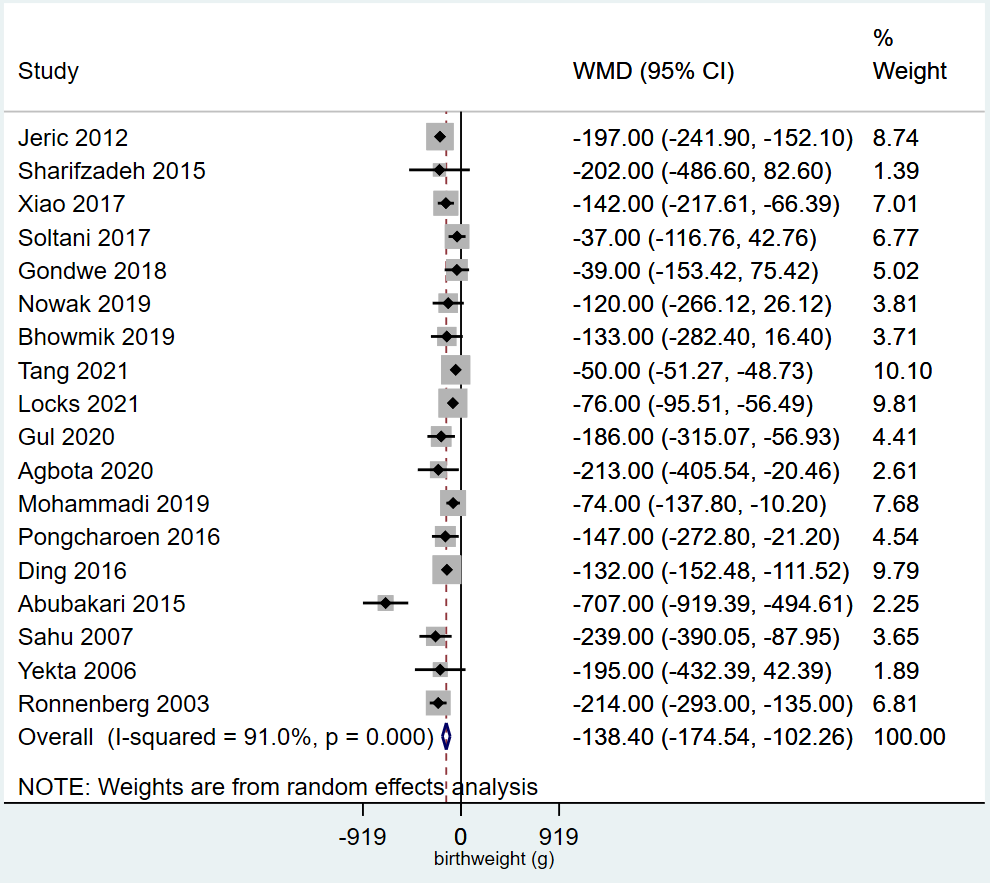 |
| WMD: weighted mean difference; CI: confidence interval; g: grams. |

| Table 3: Quality assessment of the studies included in the meta-analysis | | | | | | | | | | |
| --- | --- | --- | --- | --- | --- | --- | --- | --- | --- | --- |
|  | Selection | | | | Comparability | | Outcome | | | Study quality |
|  | 1 | 2 | 3 | 4 | 5A | 5B | 6 | 7 | 8 | Out of 7 |
| Study | **Exposed cohort truly representative** | **Nonexposed cohort drawn from the same community** | **Ascertainment of exposure** | **Outcome of interest not present at start** | **Cohorts comparable on basis of age** | **Cohorts comparable on other factor(s)** | **Quality of outcome assessment** | **Follow-up long enough for outcomes to occur** | **Complete accounting for cohorts** |  |
| Tang 2021 | 0 | 1 | 1 | 1 | NA (crude measurement desired) | NA (crude measurement desired) | 1 | 1 | 1 | 6/7 |
| Locks 2021 | 1 | 1 | 1 | 1 | NA (crude measurement desired) | NA (crude measurement desired) | 1 | 1 | 1 | 7/7 |
| Gul 2020 | 0 | 1 | 1 | 1 | NA (crude measurement desired) | NA (crude measurement desired) | 1 | 1 | 1 | 6/7 |
| Agbota 2020 | 1 | 1 | 1 | 1 | NA (crude measurement desired) | NA (crude measurement desired) | 1 | 1 | 0 | 6/7 |
| Nowak 2019 | 0 | 1 | 1 | 1 | NA (crude measurement desired) | NA (crude measurement desired) | 1 | 1 | 1 | 6/7 |
| Bhowmik 2019 | 0 | 1 | 1 | 1 | NA (crude measurement desired) | NA (crude measurement desired) | 1 | 1 | 0 | 5/7 |
| Mohammadi 2019 | 1 | 1 | 1 | 1 | NA (crude measurement desired) | NA (crude measurement desired) | 1 | 1 | 1 | 7/7 |
| Gondwe 2018 | 0 | 1 | 0 | 1 | NA (crude measurement desired) | NA (crude measurement desired) | 1 | 1 | 1 | 5/7 |
| Xiao 2017 | 0 | 1 | 0 | 1 | NA (crude measurement desired) | NA (crude measurement desired) | 1 | 1 | 1 | 5/7 |
| Soltani 2017 | 1 | 1 | 1 | 1 | NA (crude measurement desired) | NA (crude measurement desired) | 1 | 1 | 1 | 7/7 |
| Pongcharoen 2016 | 0 | 1 | 0 | 1 | NA (crude measurement desired) | NA (crude measurement desired) | 1 | 1 | 1 | 5/7 |
| Ding 2016 | 1 | 1 | 0 | 1 | NA (crude measurement desired) | NA (crude measurement desired) | 1 | 1 | 1 | 6/7 |
| Sharifzadeh 2015 | 0 | 1 | 1 | 1 | NA (crude measurement desired) | NA (crude measurement desired) | 1 | 1 | 0 | 5/7 |
| Abubakari 2015 | 0 | 1 | 1 | 1 | NA (crude measurement desired) | NA (crude measurement desired) | 1 | 1 | 1 | 6/7 |
| Jeric 2012 | 0 | 1 | 1 | 1 | NA (crude measurement desired) | NA (crude measurement desired) | 1 | 1 | 1 | 6/7 |
| Sahu 2007 | 0 | 1 | 0 | 1 | NA (crude measurement desired) | NA (crude measurement desired) | 1 | 1 | 1 | 5/7 |
| Yekta 2006 | 0 | 1 | 1 | 1 | NA (crude measurement desired) | NA (crude measurement desired) | 1 | 1 | 1 | 6/7 |
| Ronnenberg 2003 | 0 | 1 | 1 | 1 | NA (crude measurement desired) | NA (crude measurement desired) | 1 | 1 | 1 | 6/7 |

**References**

1. Yu Z, Han S, Zhu J, Sun X, Ji C, Guo X. Pre-Pregnancy Body Mass Index in Relation to Infant Birth Weight and Offspring Overweight/Obesity: A Systematic Review and Meta-Analysis. PLoS ONE. 2013;8. doi:10.1371/journal.pone.0061627

2. Rahman MM, Abe SK, Kanda M, Narita S, Rahman MS, Bilano V, et al. Maternal body mass index and risk of birth and maternal health outcomes in low- and middle-income countries: a systematic review and meta-analysis. Obes Rev. 2015;16: 758–770. doi:10.1111/obr.12293

3. Liu P, Xu L, Wang Y, Zhang Y, Du Y, Sun Y, et al. Association between perinatal outcomes and maternal pre-pregnancy body mass index. Obes Rev. 2016;17: 1091–1102. doi:10.1111/obr.12455

4. Jeric M, Roje D, Medic N, Strinic T, Mestrovic Z, Vulic M. Maternal pre-pregnancy underweight and fetal growth in relation to institute of medicine recommendations for gestational weight gain. Early Hum Dev. 2013;89: 277–281. doi:10.1016/j.earlhumdev.2012.10.004

5. Sharifzadeh F, Kashanian M, Jouhari S, Sheikhansari N. Relationship between pre-pregnancy maternal BMI with spontaneous preterm delivery and birth weight. J Obstet Gynaecol. 2015;35: 354–357. doi:10.3109/01443615.2014.968101

6. Xiao L, Ding G, Vinturache A, Xu J, Ding Y, Guo J, et al. Associations of maternal pre-pregnancy body mass index and gestational weight gain with birth outcomes in Shanghai, China. Sci Rep. 2017;7: 1–8. doi:10.1038/srep41073

7. Soltani H, Lipoeto NI, Fair FJ, Kilner K, Yusrawati Y. Pre-pregnancy body mass index and gestational weight gain and their effects on pregnancy and birth outcomes: a cohort study in West Sumatra, Indonesia. BMC Womens Health. 2017;17. doi:10.1186/s12905-017-0455-2

8. Gondwe A, Ashorn P, Ashorn U, Dewey KG, Maleta K, Nkhoma M, et al. Pre-pregnancy body mass index (BMI) and maternal gestational weight gain are positively associated with birth outcomes in rural Malawi. PLOS ONE. 2018;13: e0206035. doi:10.1371/journal.pone.0206035

9. Nowak M, Kalwa M, Oleksy P, Marszalek K, Radon-Pokracka M, Huras H. The relationship between pre-pregnancy BMI, gestational weight gain and neonatal birth weight: a retrospective cohort study. Ginekol Pol. 2019;90: 50–54. doi:10.5603/GP.2019.0008

10. Bhowmik B, Siddique T, Majumder A, Mdala I, Hossain IA, Hassan Z, et al. Maternal BMI and nutritional status in early pregnancy and its impact on neonatal outcomes at birth in Bangladesh. BMC Pregnancy Childbirth. 2019;19. doi:10.1186/s12884-019-2571-5

11. Tang J, Zhu X, Chen Y, Huang D, Tiemeier H, Chen R, et al. Association of maternal pre-pregnancy low or increased body mass index with adverse pregnancy outcomes. Sci Rep. 2021;11: 3831. doi:10.1038/s41598-021-82064-z

12. Locks LM, Patel A, Katz E, Simmons E, Hibberd P. Seasonal trends and maternal characteristics as predictors of maternal undernutrition and low birthweight in Eastern Maharashtra, India. Matern Child Nutr. 2021;17: e13087. doi:10.1111/mcn.13087

13. Gul R, Iqbal S, Anwar Z, Ahdi SG, Ali SH, Pirzada S. Pre-pregnancy maternal BMI as predictor of neonatal birth weight. PloS One. 2020;15: e0240748. doi:10.1371/journal.pone.0240748

14. Agbota G, Fievet N, Heude B, Accrombessi M, Ahouayito U, Yovo E, et al. Poor maternal anthropometric status before conception is associated with a deleterious infant growth during the first year of life: a longitudinal preconceptional cohort. Pediatr Obes. 2020;15: e12573. doi:10.1111/ijpo.12573

15. Mohammadi M, Maroufizadeh S, Omani-Samani R, Almasi-Hashiani A, Amini P. The effect of prepregnancy body mass index on birth weight, preterm birth, cesarean section, and preeclampsia in pregnant women. J Matern Fetal Neonatal Med. 2019;32: 3818–3823. doi:10.1080/14767058.2018.1473366

16. Pongcharoen T, Gowachirapant S, Wecharak P, Sangket N, Winichagoon P. Pre-pregnancy body mass index and gestational weight gain in Thai pregnant women as risks for low birth weight and macrosomia. Asia Pac J Clin Nutr. 2016;25: 810–817. doi:10.6133/apjcn.092015.41

17. Ding X-X, Xu S-J, Hao J-H, Huang K, Su P-Y, Tao F-B. Maternal pre-pregnancy BMI and adverse pregnancy outcomes among Chinese women: Results from the C-ABCS. J Obstet Gynaecol. 2016;36: 328–332. doi:10.3109/01443615.2015.1050652

18. Abubakari A, Kynast-Wolf G, Jahn A. Maternal Determinants of Birth Weight in Northern Ghana. Meyre D, editor. PLOS ONE. 2015;10: e0135641. doi:10.1371/journal.pone.0135641

19. Sahu MT, Agarwal A, Das V, Pandey A. Impact of maternal body mass index on obstetric outcome. J Obstet Gynaecol Res. 2007;33: 655–659. doi:10.1111/j.1447-0756.2007.00646.x

20. Yekta Z, Ayatollahi H, Porali R, Farzin A. The effect of pre-pregnancy body mass index and gestational weight gain on pregnancy outcomes in urban care settings in Urmia-Iran. BMC Pregnancy Childbirth. 2006;6: 15–8. doi:10.1186/1471-2393-6-15

21. Ronnenberg AG, Wang X, Xing H, Chen C, Chen D, Guang W, et al. Low preconception body mass index is associated with birth outcome in a prospective cohort of Chinese women. J Nutr. 2003;133: 3449–3455. doi:10.1093/jn/133.11.3449
