# Supplementary material for: Cost-effectiveness of antenatal multiple micronutrients and balanced energy protein supplementation compared to iron and folic acid supplementation in India, Pakistan, Mali, and Tanzania: A dynamic microsimulation study
Source: PLoS Med. 2022 Feb 22;19(2):e1003902. doi: 10.1371/journal.pmed.1003902 (PMC8863292; doi:10.1371/journal.pmed.1003902)
Supplement: S3 Supplement — (DOCX) [file pmed.1003902.s003.docx]

**SUPPLEMENT 3**

**Meta-analysis of birthweight shift of multiple micronutrients vs. iron with or without folic acid control**

In order to model the effect of multiple micronutrient (MMS) supplementation of the pregnant mother on infant birthweight, we need to know the birthweight difference between those supplemented with MMS and those supplemented with iron and folic acid (IFA). The Cochrane review by Keats et al. (2019) analyzed 18 trials and found a reduced risk of low birthweight among those supplemented with MMS and IFA vs. just iron with or without folic acid (pooled risk ratio 0.88 (95%UI 0.85-0.91)). We reviewed these 18 trials and extracted estimates of mean birthweight shifts when reported to conduct our own meta-analysis of mean differences. Thirteen of 18 studies reported mean shifts.

Table 1: Mean and standard deviation of birthweight among mothers supplemented with multiple micronutrients (MMS) with iron and folic acid and mothers supplemented with iron with or without folic acid from individual trials with sufficient data included in the Keats et al. (2019) Cochrane review.

| **Study** | **MMS with iron and folic acid** | | **Iron with or without folic acid** | |
| --- | --- | --- | --- | --- |
|  | **N** | **Mean (SD)** | **N** | **Mean (SD)** |
| Friis 2004 [1] | 564 | 3053 (500^†^) | 542 | 3004 (500^†^) |
| Osrin 2005 [2] | 600 | 2810 (453) | 600 | 2733 (422) |
| Bhutta 2009 [3] | 1,148 | 2950 (600) | 1,230 | 2880 (500) |
| Christian 2003 [4] | 1,038 | 2659 (446) | 940 | 2652 (436) |
| Dewey 2009 [5] | 354 | 3005 (435) | 349 | 2945 (442) |
| Kaestel 2005 [6] | 360 | 3055 (483*) | 366 | 3002 (532*) |
| Ramakrishnan 2003 [7] | 435 | 2981 (391) | 438 | 2977 (393) |
| Roberfroid 2008 [8] | 526 | 2914 (450) | 526 | 2877 (424) |
| SUMMIT 2008 [9] | 5,695 | 3198 (847*) | 5,406 | 3176 (862*) |
| Sunawang 2009 [10] | 384 | 3094 (438) | 341 | 3054 (419) |
| West 2014 [11] | 10,642 | 2585 (407) | 10,530 | 2531 (415) |
| Zagre 2007 [12] | 1,328 | 3,092 (190) | 1,222 | 3,025 (205) |
| Zeng 2008 [13] | 1,406 | 3,198 (438) | 1,470 | 3,174 (424) |
| † No standard deviations were reported, so used the SD they used for their sample size calculation  *SDs were derived from 95%CIs with equation: 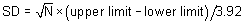 | | | | |

**Figure 1: Forest plot of mean difference in birthweight between multi-micronutrients (MMS) with iron and folic acid vs control**

| 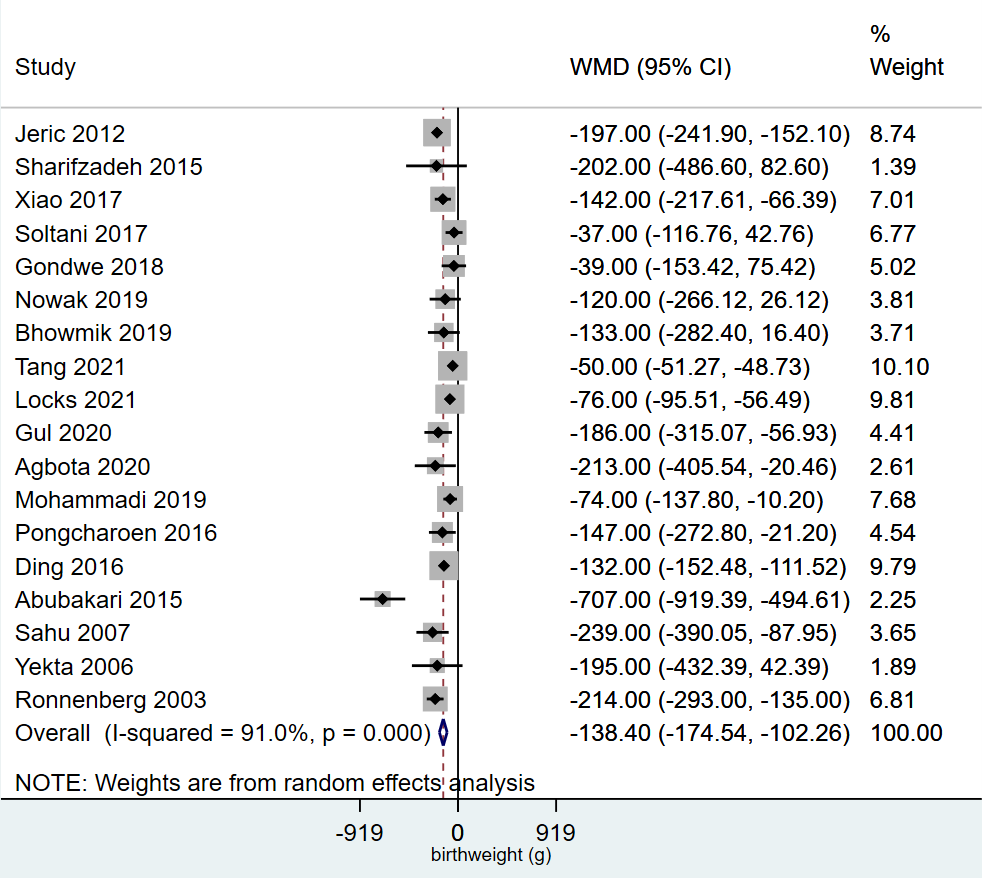  WMD: weighted mean difference; CI: confidence interval; g: grams. |
| --- |

**References**

1 Friis H, Gomo E, Nyazema N, *et al.* Effect of multimicronutrient supplementation on gestational length and birth size: a randomized, placebo-controlled, double-blind effectiveness trial in Zimbabwe. *Am J Clin Nutr* 2004; **80**: 178–84.

2 Osrin D, Vaidya A, Shrestha Y, *et al.* Effects of antenatal multiple micronutrient supplementation on birthweight and gestational duration in Nepal: double-blind, randomised controlled trial. *Lancet Lond Engl* 2005; **365**: 955–62.

3 Bhutta ZA, Rizvi A, Raza F, *et al.* A comparative evaluation of multiple micronutrient and iron-folic acid supplementation during pregnancy in Pakistan: impact on pregnancy outcomes. *Food Nutr Bull* 2009; **30**: S496-505.

4 Christian P, Khatry SK, Katz J, *et al.* Effects of alternative maternal micronutrient supplements on low birth weight in rural Nepal: double blind randomised community trial. *BMJ* 2003; **326**: 571.

5 Adu-Afarwuah S, Lartey A, Okronipa H, *et al.* Lipid-based nutrient supplement increases the birth size of infants of primiparous women in Ghana. *Am J Clin Nutr* 2015; **101**: 835–46.

6 Kaestel P, Michaelsen KF, Aaby P, Friis H. Effects of prenatal multimicronutrient supplements on birth weight and perinatal mortality: a randomised, controlled trial in Guinea-Bissau. *Eur J Clin Nutr* 2005; **59**: 1081–9.

7 Ramakrishnan U, González-Cossío T, Neufeld LM, Rivera J, Martorell R. Multiple micronutrient supplementation during pregnancy does not lead to greater infant birth size than does iron-only supplementation: a randomized controlled trial in a semirural community in Mexico. *Am J Clin Nutr* 2003; **77**: 720–5.

8 Roberfroid D, Huybregts L, Lanou H, *et al.* Effects of maternal multiple micronutrient supplementation on fetal growth: a double-blind randomized controlled trial in rural Burkina Faso. *Am J Clin Nutr* 2008; **88**: 1330–40.

9 Supplementation with Multiple Micronutrients Intervention Trial (SUMMIT) Study Group, Shankar AH, Jahari AB, *et al.* Effect of maternal multiple micronutrient supplementation on fetal loss and infant death in Indonesia: a double-blind cluster-randomised trial. *Lancet Lond Engl* 2008; **371**: 215–27.

10 Sunawang null, Utomo B, Hidayat A, Kusharisupeni null, Subarkah null. Preventing low birthweight through maternal multiple micronutrient supplementation: a cluster-randomized, controlled trial in Indramayu, West Java. *Food Nutr Bull* 2009; **30**: S488-495.

11 West KP, Shamim AA, Mehra S, *et al.* Effect of maternal multiple micronutrient vs iron-folic acid supplementation on infant mortality and adverse birth outcomes in rural Bangladesh: the JiVitA-3 randomized trial. *JAMA* 2014; **312**: 2649–58.

12 Zagré NM, Desplats G, Adou P, Mamadoultaibou A, Aguayo VM. Prenatal multiple micronutrient supplementation has greater impact on birthweight than supplementation with iron and folic acid: a cluster-randomized, double-blind, controlled programmatic study in rural Niger. *Food Nutr Bull* 2007; **28**: 317–27.

13 Zeng L, Dibley MJ, Cheng Y, *et al.* Impact of micronutrient supplementation during pregnancy on birth weight, duration of gestation, and perinatal mortality in rural western China: double blind cluster randomised controlled trial. *BMJ* 2008; **337**: a2001.
