# Supplementary material for: Cost-effectiveness of antenatal multiple micronutrients and balanced energy protein supplementation compared to iron and folic acid supplementation in India, Pakistan, Mali, and Tanzania: A dynamic microsimulation study
Source: PLoS Med. 2022 Feb 22;19(2):e1003902. doi: 10.1371/journal.pmed.1003902 (PMC8863292; doi:10.1371/journal.pmed.1003902)
Supplement: S5 Supplement — (DOCX) [file pmed.1003902.s005.docx]

**SUPPLEMENT 5**

**Model verification plots comparing Global Burden of Disease Study inputs and simulation** **outputs**

| **Figure 1: Model verification of the age- and location-specific all-cause mortality rate, defined as deaths per 100,000 person-years, using the 2017 Global Burden of Disease Study model inputs and simulation model outputs with 95% uncertainty intervals** |
| --- |
| **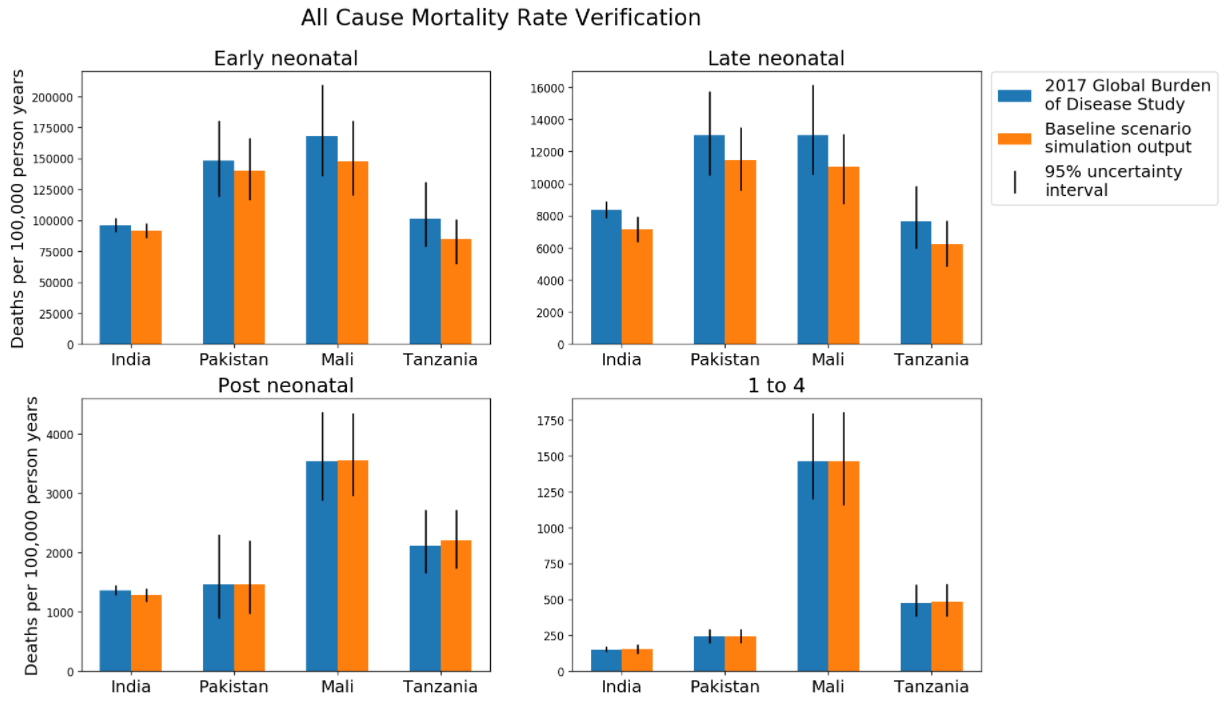**  Early neonatal: 0-6 days; late neonatal: 7-27 days; post neonatal: 28-364 days; 1 to 4: 1-4 years.  **Figure 2: Model verification for diarrheal diseases in the first two years of life using the 2017 Global Burden of Disease Study model inputs and simulation model outputs with 95% uncertainty intervals.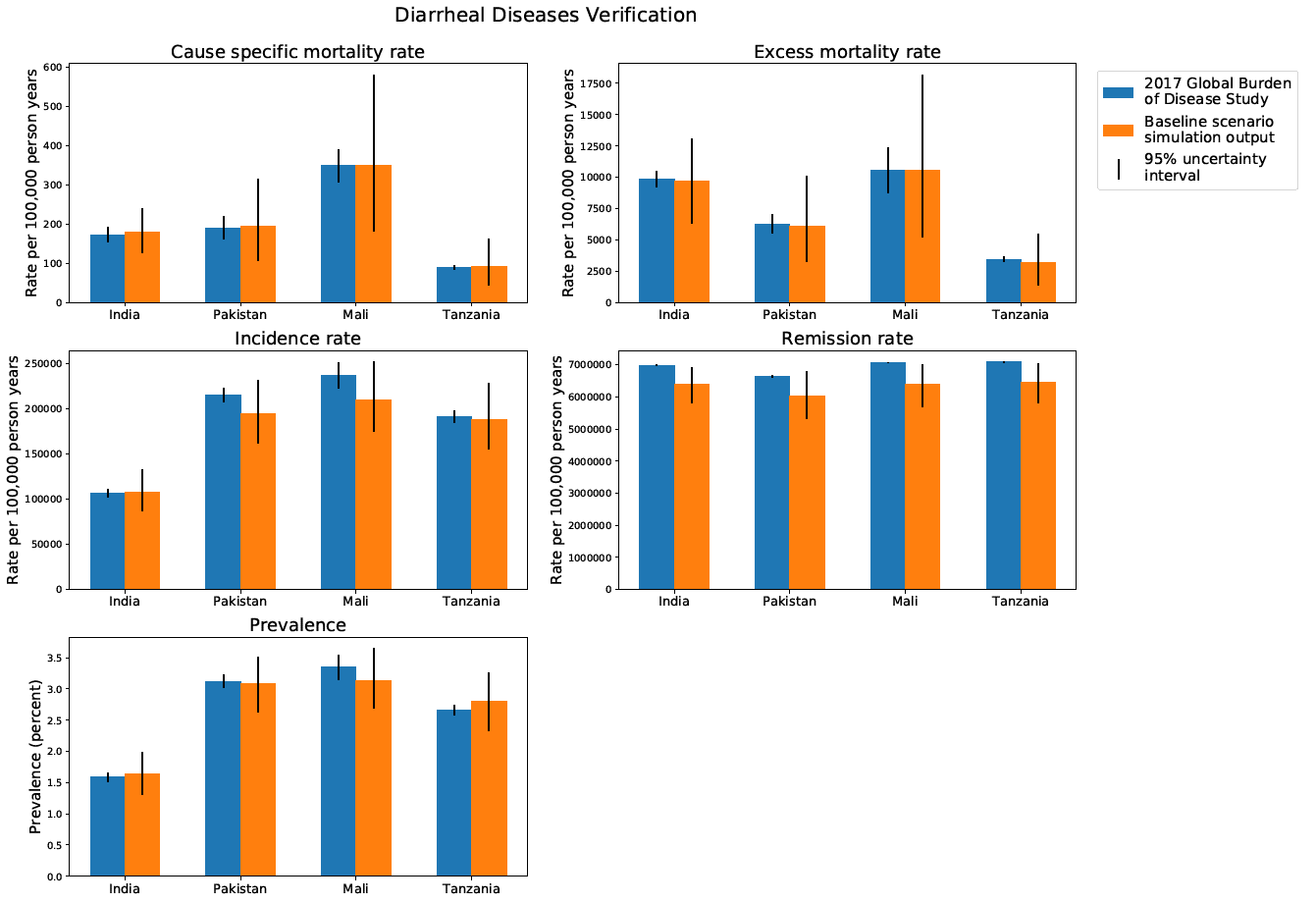**  Cause-specific mortality rate: deaths due to diarrheal diseases per 100,000 person-years among children under two. Excess mortality rate: deaths due to diarrheal diseases per 100,000 person-years among children under two with diarrheal diseases. Incidence rate: incidence cases of diarrheal diseases per 100,000 person-years among children under two. Remission rate: remitted cases of diarrheal diseases per 100,000 person-years among children under two with diarrheal diseases. Prevalence: percent of the under two population with diarrheal diseases at a point in time.    **Figure 3: Model verification for lower respiratory infections in the first two years of life using the 2017 Global Burden of Disease Study model inputs and simulation model outputs with 95% uncertainty intervals.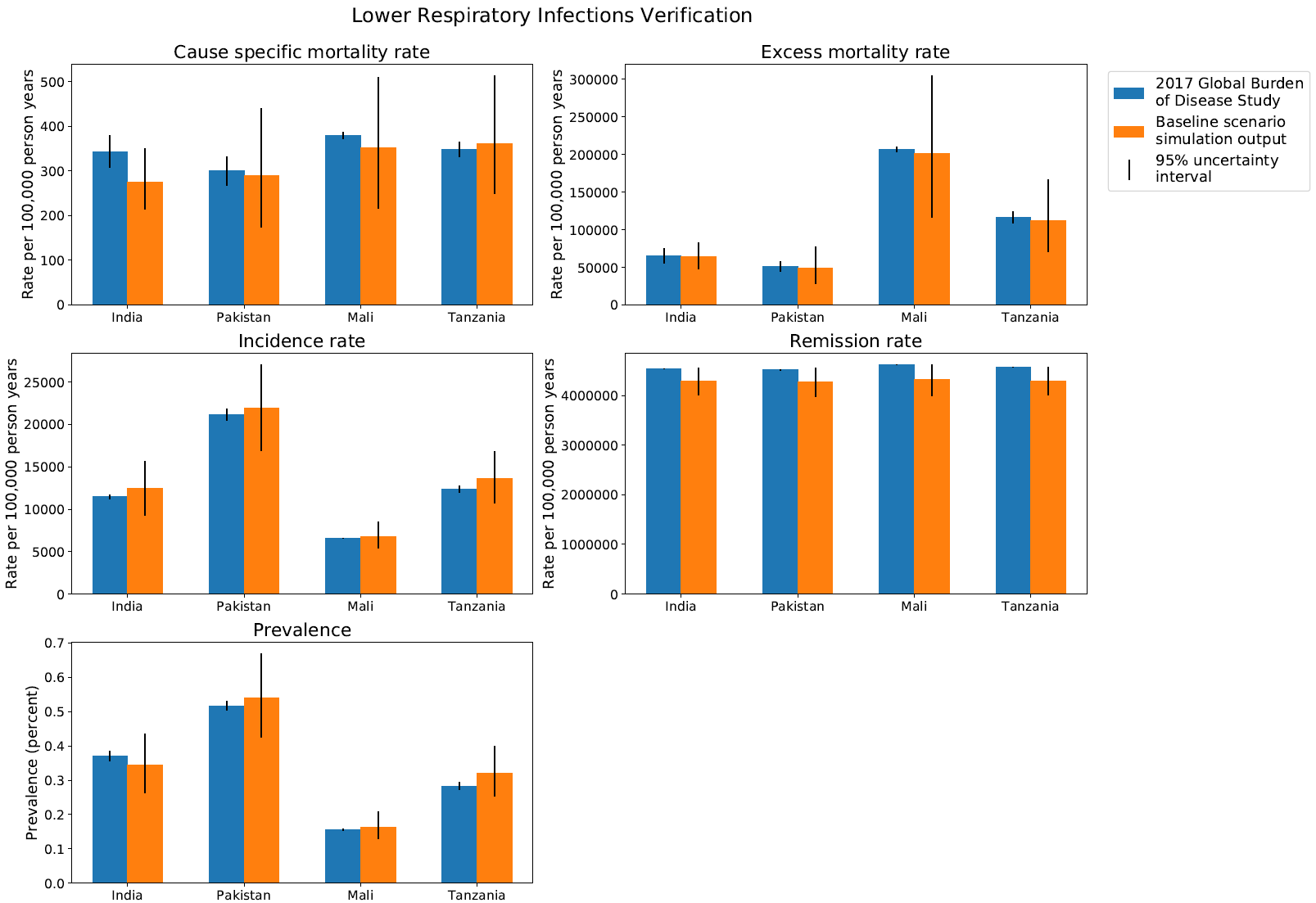**  Cause-specific mortality rate: deaths due to lower respiratory infections per 100,000 person-years among children under two. Excess mortality rate: deaths due to lower respiratory infections per 100,000 person-years among children under two with lower respiratory infections. Incidence rate: incidence cases of lower respiratory infections per 100,000 person-years among children under two. Remission rate: remitted cases of lower respiratory infections per 100,000 person-years among children under two with lower respiratory infections. Prevalence: percent of the under two population with lower respiratory infections at a point in time.  **Figure 4: Model verification for measles in the first two years of life using the 2017 Global Burden of Disease Study model inputs and simulation model outputs with 95% uncertainty intervals.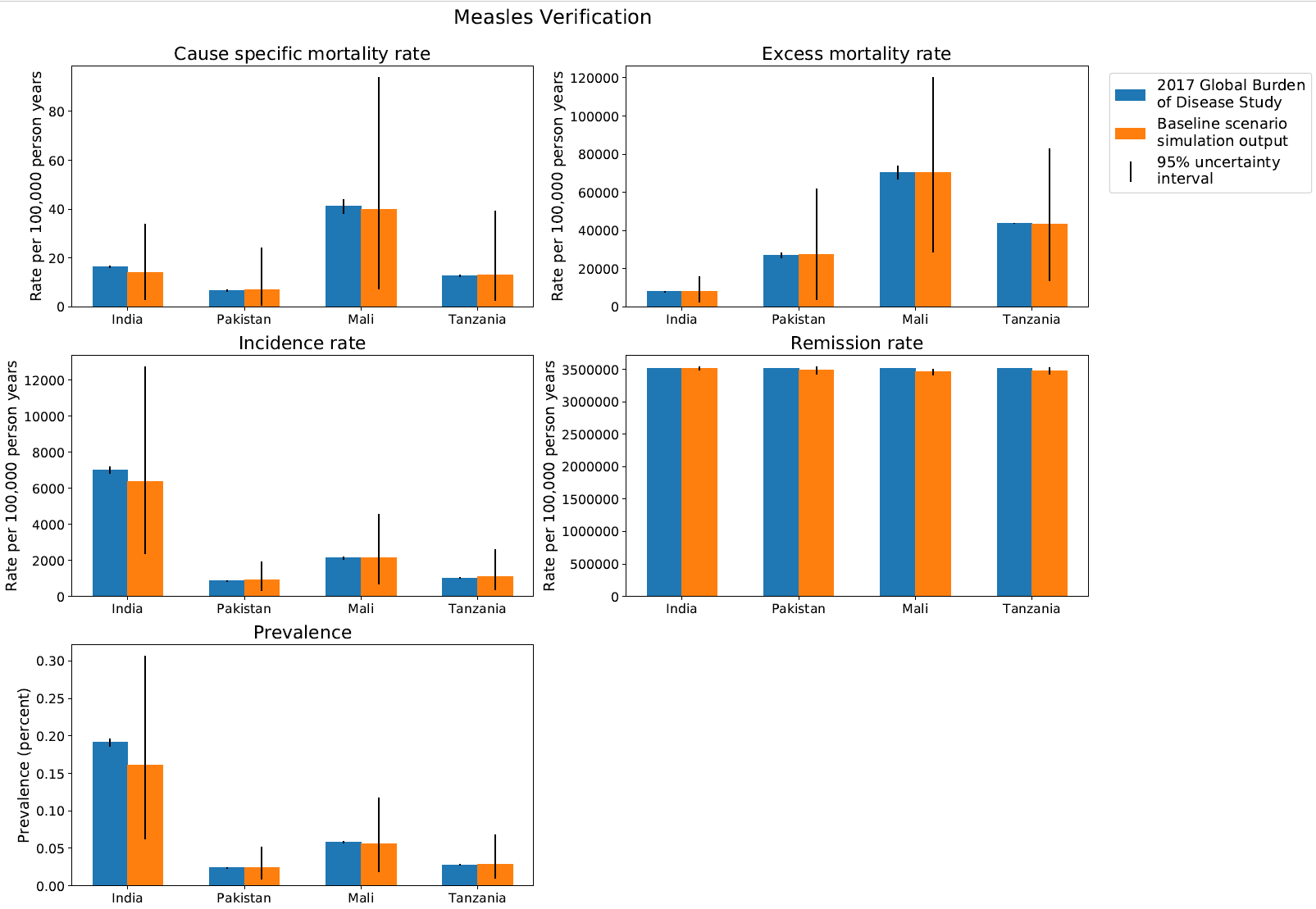**  Cause-specific mortality rate: deaths due to measles per 100,000 person-years among children under two. Excess mortality rate: deaths due to measles per 100,000 person-years among children under two with measles. Incidence rate: incidence cases of measles per 100,000 person-years among children under two. Remission rate: remitted cases of measles per 100,000 person-years among children under two with measles. Prevalence: percent of the under two population with measles at a point in time**.**  **Figure 5: Model verification for protein energy malnutrition in the first two years of life using the 2017 Global Burden of Disease Study model inputs and simulation model outputs with 95% uncertainty intervals.**  **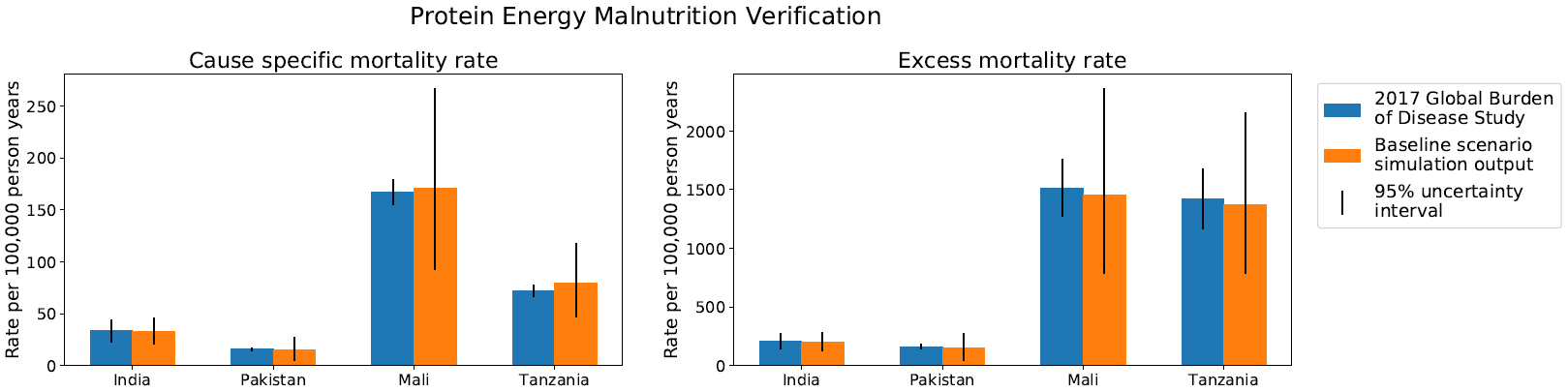**  Cause-specific mortality rate: deaths due to protein energy malnutrition per 100,000 person-years among children under two. Excess mortality rate: deaths due to protein energy malnutrition per 100,000 person-years among children under two with protein energy malnutrition. |
