# Supplementary material for: Cost-effectiveness of antenatal multiple micronutrients and balanced energy protein supplementation compared to iron and folic acid supplementation in India, Pakistan, Mali, and Tanzania: A dynamic microsimulation study
Source: PLoS Med. 2022 Feb 22;19(2):e1003902. doi: 10.1371/journal.pmed.1003902 (PMC8863292; doi:10.1371/journal.pmed.1003902)
Supplement: S6 Supplement — (DOCX) [file pmed.1003902.s006.docx]

**SUPPLEMENT 6**

**Sensitivity Analysis Figures**

| **Figure 1: Incremental cost and disability adjusted life years (DALYs) averted among the first two years of life per 100,000 births for each low coverage sensitivity analysis simulated intervention scenario relative to the baseline scenario in each modeled location per 100,000 live births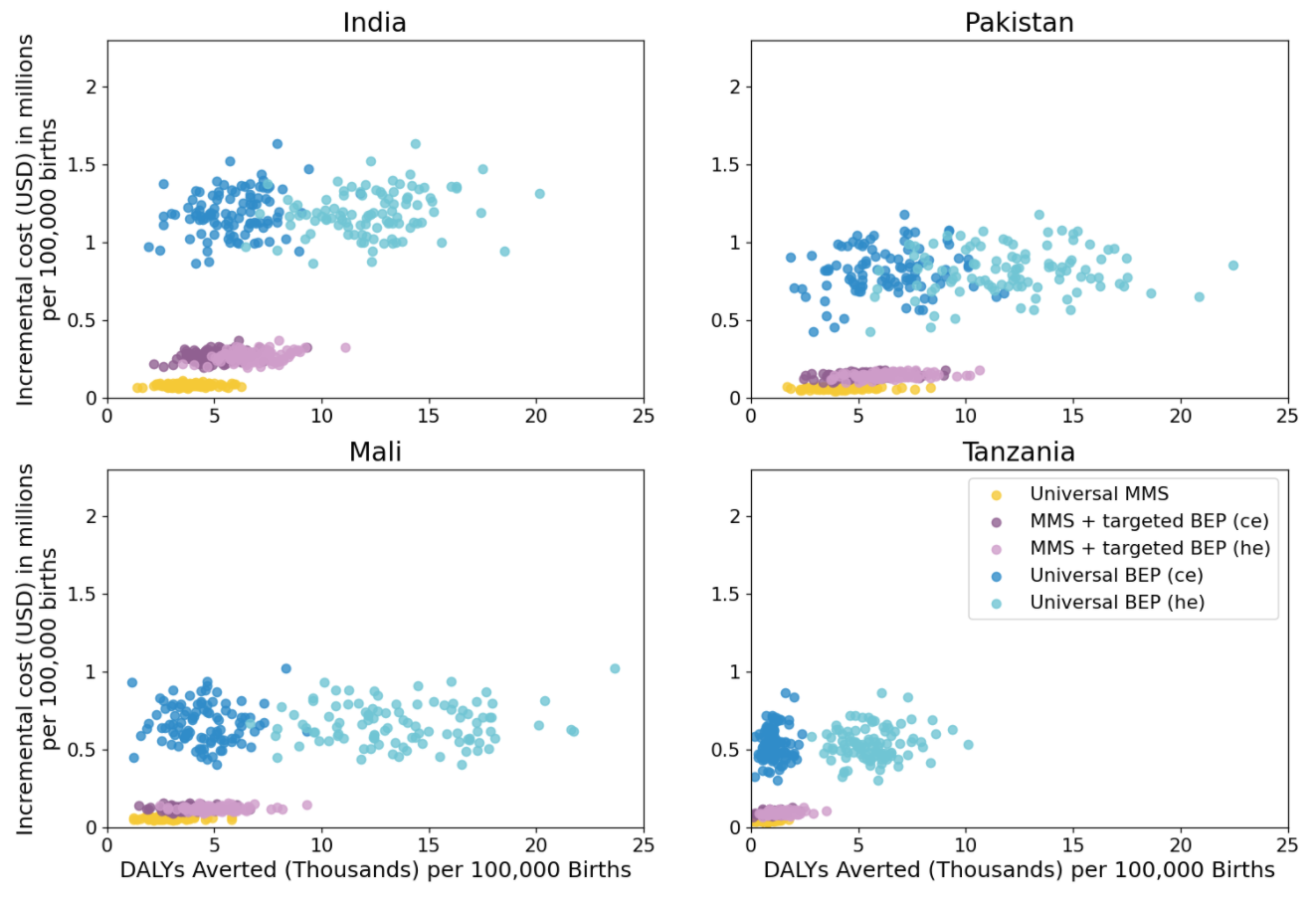**  MMS: multiple micronutrient supplementation; BEP: balanced-energy protein  ce- scenarios with current evidence effects for BEP; he-scenarios with hypothesized evidence effects for BEP  *Each point in this figure represents one of the 100 simulation runs performed, each of which used varying parameter values within the parameter uncertainty intervals used in our model. |
| --- |

| **Figure 2: Incremental cost per disability adjusted life year (DALY) averted among the first two years of life in each modeled location for each intervention coverage sensitivity analysis scenario relative to the baseline scenario (log-scale)** |
| --- |
| 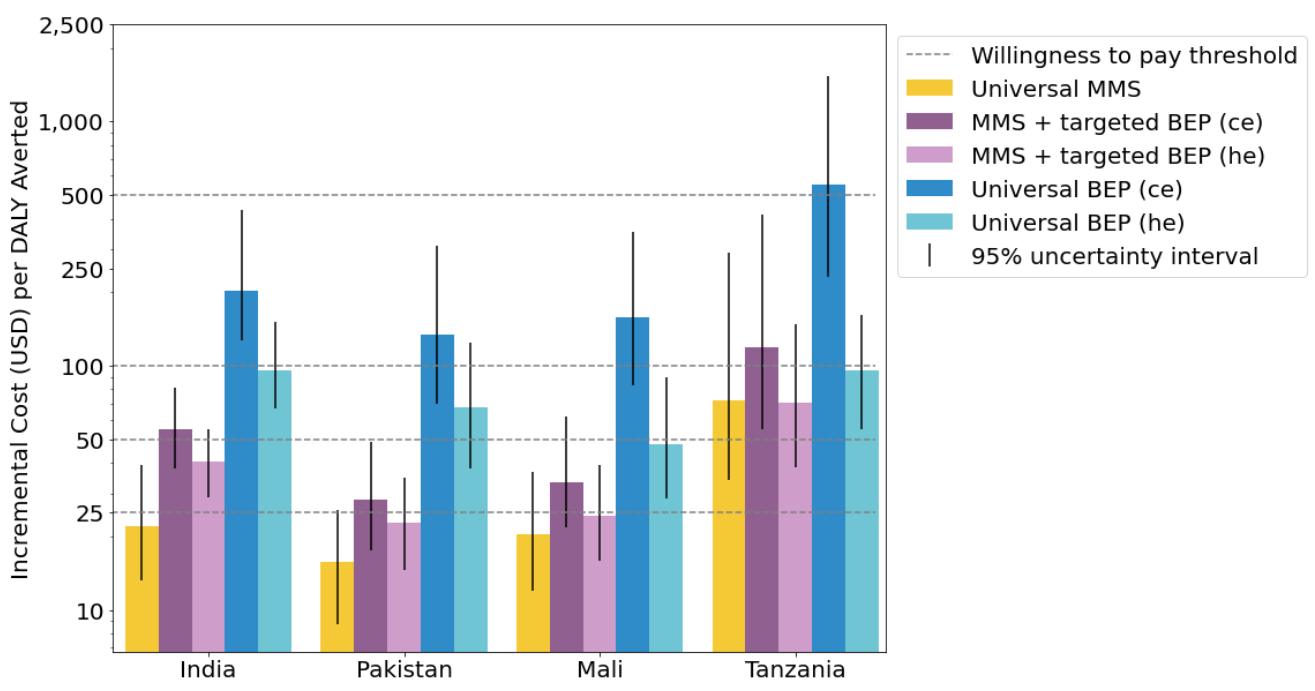 |
| MMS: multiple micronutrient supplementation; BEP: balanced-energy protein  ce- scenarios with current evidence effects for BEP; he-scenarios with hypothesized evidence effects for BEP |
